# mtDNA T8993G Mutation-Induced F1F0-ATP Synthase Defect Augments Mitochondrial Dysfunction Associated with hypoxia/reoxygenation: The Protective Role of Melatonin

**DOI:** 10.1371/journal.pone.0081546

**Published:** 2013-11-29

**Authors:** Wen-Yi Huang, Mei-Jie Jou, I. Peng Tsung

**Affiliations:** 1 Graduate Institute of Clinical Medical Sciences, College of Medicine, Chang Gung University, Tao-Yuan, Taiwan; 2 Department of Neurology, Chang-Gung Memorial Hospital, Keelung Branch, Taiwan; 3 Department of Physiology and Pharmacology, College of Medicine, Chang Gung University, Tao-Yuan, Taiwan; 4 Department of Medicine, College of Medicine, Chang Gung University, Tao-Yuan, Taiwan; University of Texas Health Science Center at San Antonio, United States of America

## Abstract

**Background:**

F1F0-ATP synthase (F1F0-ATPase) plays important roles in regulating mitochondrial function during hypoxia, but the effect of F1F0-ATPase defect on hypoxia/reoxygenation (H/RO) is unknown. The aim of this study was to investigate how mtDNA T8993G mutation (NARP)-induced inhibition of F1F0-ATPase modulates the H/RO–induced mitochondrial dysfunction. In addition, the potential for melatonin, a potent antioxidant with multiple mitochondrial protective properties, to protect NARP cells exposed to H/RO was assessed.

**Methods And Findings:**

NARP cybrids harboring 98% of mtDNA T8993G genes were established as an in vitro model for cells with F1F0-ATPase defect; their parental osteosarcoma 143B cells were studied for comparison. Treating the cells with H/RO using a hypoxic chamber resembles ischemia/reperfusion in vivo. NARP significantly enhanced apoptotic death upon H/RO detected by MTT assay and the trypan blue exclusion test of cell viability. Based on fluorescence probe-coupled laser scanning imaging microscopy, NARP significantly enhanced mitochondrial reactive oxygen species (mROS) formation and mitochondrial Ca^2+^ (mCa^2+^) accumulation in response to H/RO, which augmented the depletion of cardiolipin, resulting in the retardation of mitochondrial movement. With stronger H/RO stress (either with longer reoxygenation duration, longer hypoxia duration, or administrating secondary oxidative stress following H/RO), NARP augmented H/RO-induced mROS formation to significantly depolarize mitochondrial membrane potential (ΔΨm), and enhance mCa^2+^ accumulation and nitric oxide formation. Also, NARP augmented H/RO-induced mROS oxidized and depleted cardiolipin, thereby promoting permanent mitochondrial permeability transition, retarded mitochondrial movement, and enhanced apoptosis. Melatonin markedly reduced NARP-augmented H/RO-induced mROS formation and therefore significantly reduced mROS-mediated depolarization of ΔΨm and accumulation of mCa^2+^, stabilized cardiolipin, and then improved mitochondrial movement and cell survival.

**Conclusion:**

NARP-induced inhibition of F1F0-ATPase enhances mROS formation upon H/RO, which augments the depletion of cardiolipin and retardation of mitochondrial movement. Melatonin may have the potential to rescue patients with ischemia/reperfusion insults, even those associated with NARP symptoms.

##  Introduction

Tissue ischemia, such as acute cerebral or myocardial infarction, is characterized by severe hypoxia, acidosis, energy depletion, and cell death. Although the timely restoration of blood flow, such as infusion with tissue plasminogen activator (t-PA) or intra-arterial thrombolysis, have proven to be the most effective therapies for minimizing ischemic injury, reperfusion of ischemic tissue can result in harmful consequences [1,2]. Currently the mechanism of this hypoxia/reoxynegation (H/RO) injury remains uncertain. It has been shown that prolonged hypoxia damage mitochondria and inhibit the activity of electron transport chain, and proton pumping across the inner mitochondrial membrane (IMM) are inhibited, leading to ATP depletion, intracellular acidification, and Ca^2+^ overload [3-8]. The damaged mitochondria are no longer able to efficiently transfer electrons at reoxygenation, thereby greatly increasing reactive oxygen species (ROS) generation from complexes-I and –III [9-13]. The excessive ROS formation, Ca^2+^ overload and recovery of pH value induce the abrupt opening of the mitochondrial permeability transition pores (mPTP), which strongly contributes to cell death [14-17]. Thus excessive ROS formation in mitochondria is regarded as a crucial contributor of H/RO injury [1,18,19]. 

F1F0-ATP synthase (F1F0-ATPase) is the enzyme responsible for catalyzing ADP phosphorylation in oxidative phosphorylation (OXPHOS) by using the proton motive force across the IMM to drive the synthesis of ATP. To the best of our knowledge, the effect of F1F0-ATPase defect on H/RO injury has not been previously studied. Among human inherited mitochondrial disorders, the mtDNA T8993G mutation (Leu156Arg), or NARP, is well known to result in the potent inhibition of ATPase 6 of F1F0-ATPases and severe ATP deficiency [20]. Recently, our group had identified the mitochondrial characters of NARP cybrids cells (cells with 98% mtDNA T8993G mutation) in response to several apoptotic insults [21]. It has shown that NARP mutation potentiates cell apoptosis by augmenting mitochondrial ROS (mROS) formation, either in resting levels or in response to apoptotic insults (H_2_O_2_). Enhanced production of mROS affects DNA, enzymes and phospholipids (e.g., cardiolipin), which results in further abnormalities in mitochondrial function and exacerbates the pathology in NARP cybrids cells [22]. In addition, the mitochondrial membrane potential (ΔΨm) of NARP cybrids cells is more hyperpolarized at rest but is more vulnerable to the oxidative insult (H_2_O_2_) than that in wild-type cells [21]. This cell model provides a good opportunity to survey the influence of the F1F0-ATPase defect on H/RO injury.

Many recent publications present evidence that melatonin and several of its metabolites have significant protective actions against H/RO injury [23-27]. Melatonin add on t-PA infusion could rescue t-PA-induced H/RO injury in focal cerebral ischemia of mice [28,29]. By stabilizing cardiolipin, a unique mitochondrial protective phospholipid localized almost exclusively within the IMM, and preventing its oxidization and depletion, melatonin can rescue the retardation of mitochondrial movement, mitochondrial fission and swelling upon several apoptotic insults [21]. However, whether NARP-induced inhibition of F1F0-ATPase disrupts the protective effects by melatonin in response to H/RO insults is unclear. Here we found NARP-induced inhibition of F1F0-ATPase augmented H/RO insults-induced mROS formation, mitochondrial Ca^2+^(mCa^2+^) accumulation, ΔΨm depolarization, cardiolipin depletion, and mitochondrial movement retardation, eventually increasing cell apoptosis. The administration of melatonin modulated these mitochondrial dysfunctions, and rescued either H/RO-induced or NARP-related cell apoptosis. These findings indicate important insight of the protective effect of melatonin in H/RO injury, lighting a new neuroprotective strategy during H/RO injury. 

## Materials and Methods

### Establishment of NARP Cybrids

The NARP cybrids were established as described previously [30]. Briefly, skin fibroblasts obtained from a patient with Leigh’s disease carrying the mtDNA T8993G mutation were enucleated and cytoplasmically fused with mtDNA-less (ρ°) human osteosarcoma 143B cells. The NARP cybrids and ρ° cells were maintained in Dulbecco’s modified Eagle’s medium (DMEM) containing 10% fetal bovine serum supplemented with high glucose (4.5 g/mL), pyruvate (0.11 mg/mL), and uridine (0.1 mg/mL). NARP cybrids with a high mutant mtDNA to wild-type mtDNA ratio of approximately 98% were used for experiments, and comparisons were made with the parental 143B cell line. Both the NARP cybrids and 143B cells described above were kindly provided by Dr. Tanaka from Japan [48]. The cells had been used in other previous studies by our group [28,52]. 

### Hypoxia/reoxygenation Treatment to NARP Cybrids and 143B Cells

All cell cultures were obtained by plating at low density in DMEM + 10% FBS. All cell types were used after 48–72 h in culture. To induce hypoxia, cell cultures were put in a modular incubator chamber flushed with the gas mixture of 5% CO_2_ and 95% N_2_ for 20 min according to the manufacturer’s instructions (Billups-Rothenberg, Inc., Del Mar, CA). The deoxygenation reagent (5% CO_2_ and <1% O_2_; Mitsubishi Gas Chemical, Tokyo, Japan) was placed inside the chamber. Next, the sealed chamber was placed into a 37°C incubator. The chamber was incubated for different hypoxic durations (6, 12, and 18 h). After hypoxia incubation, the cells were washed with normoxic culture medium twice, and then transferred to their respective normal culture medium and restored to the 37°C incubator with 5% CO_2_ for reoxygenation (1, 2, 3, and 4 h). As reoxygenation after hypoxia results in overproduction of mROS, a secondary oxidative stress (5 mM H_2_O_2_) was administered following H/RO treatment in some experiments for augmenting and accelerating the effect of H/RO insults. For melatonin treatment during H/RO treatment, 100 μm melatonin (Sigma Aldrich, St. Louis, MO) was added to all media and buffers during the whole procedure of H/RO treatment and 100 μm melatonin was also added in 5 mM H_2_O_2_ if used. For supplemental data, mitochondria-specific antioxidant MitoQ (0.2 nM) or general antioxidant vitamin E (200 μm) was added to all media and buffers during the whole procedure of H_2_O_2_-augmented H/RO treatment.

### Measurement of Cell Viability

Cell viability was detected by using the colorimetric 3-(4,5-dimethyl-2-thiazolyl)-2,5-diphenyl-2H-tetrazolium bromide (MTT) assay as previously described [31]. The activity of the mitochondrial reductase to convert a soluble tetrazolium salt into an insoluble formazan precipitate was measured using an enzyme-linked immunoabsorbent assay (ELISA) reader (A-5082; TECAN, Grödig/Salzburg, Austria). The MTT assay was performed 1 h after stress exposure. The activity of the mitochondrial reductase was calculated as the amount of MTT dye conversion in treated cells relative to that of sham-treated control cells. Data are represented as the means ± standard error (SE) of at least three independent experiments. In addition, we used the trypan blue exclusion test of cell viability as previously described to measure cell viability in response to H/RO treatment [32].

### Apoptotic Cell Analysis

The flip-flop of phosphatidylserine (PS) from the inner- to the outer-plasma-membrane leaflet is a common phenomenon in apoptosis. The exposure of PS is an early event that precedes cell shrinkage and nuclear condensation. PS exposure induced by H/R in cells was detected by FITC-conjugated Annexin V-FITC staining [33]. This is observed as green fluorescence on the plasma membrane during the occurrence of PS externalization. The precise time point for PS exposure and cell death were carefully detected by the imaging of cells dual-labeled with Annexin V-FITC and propidium iodide (PI) after they were exposed to H/RO treatment using fluorescence microscopy.

### Immunocytochemical Analysis for Detecting Cytochrome c Release from Mitochondria into the Cytosol after Hypoxia/Reoxygenation

Apoptotic events were identified by cytochrome *c* distribution after H/RO. Cells were grown on #1 glass cover slips for 48 h in DMEM containing 10% fetal bovine serum supplemented with high glucose (4.5 g/mL), pyruvate (0.11 mg/mL), and uridine (0.1 mg/mL). After H/RO, cells were rinsed with phosphate buffered saline (PBS), and then fixed in 3.7% paraformaldehyde for 15 min at room temperature (RT). After fixation, the cover slips were rinsed in PBS and placed in 0.1% Triton X-100 for 10 min at RT. The cells were then washed with PBS and incubated with 1% bovine serum albumin (BSA) for 1 h. For immunostaining of mitochondrial complex II and cytochrome *c*, glass cover slips were incubated with primary antibodies (mouse) diluted 1:100 in PBST for 1 h at RT, and then the cover slips were washed 3 times for 5 min each in PBS. Cover slips were first incubated with a secondary antibody, tetramethyl rhodamine rabbit anti-mouse antibody (Acris antibody) diluted at 1:1000 in phosphate buffered saline (PBS) for 60 min at RT, and then, the cover slips were further incubated for 1 h at RT. After the cover slips were washed 3 times, they were incubated with another primary antibody (rabbit) diluted at 1:100 in PBS-Tween (PBST) for 1 h at RT. Then the cover slips were washed 3 times for 5 min each in PBS. The cover slips were then exposed for 60 min at RT to an Alexa fluor^®^ 488 conjugated goat anti-rabbit IgG (H+L) secondary antibody (Abcam, Cambridge, MA, USA) diluted 1:1000. The cover slips were washed 3 times for 5 min in PBS and then incubated for 1 min with PBS containing Hoechst 33342 (1 μg/mL), and then washed with PBS (3 washes 5 min each) before mounting for observation. The fluorescence intensity of complex expression was observed under a Zeiss inverted microscope.

### Cell Preparation for Imaging

For imaging detection, cells were grown in medium consisting of DMEM containing 10% fetal bovine serum supplemented with high glucose (4.5 g/mL), pyruvate (0.11 mg/mL), and uridine (0.1 mg/mL). All cells were plated onto #1 glass cover slips for fluorescent microscopy.

### Chemical and Fluorescent Dye Loading for Fluorescence Measurement of Mitochondrial Events

All chemicals were obtained from Sigma-Aldrich and fluorescent dyes were purchased from Molecular Probes Inc. (Eugene, OR, USA). Loading conditions for each specific fluorescent probe are described as follows: ΔΨm was detected using 200 nM tetramethylrhodamine methyl ester (TMRM); mCa^2+^ was detected using 2 μM Rhod-2 AM (Rhod-2); ROS was detected using 2 μM 6-carboxy-2′,7′-dichlorodihydrofluorescein diacetate (DCFH-DA); nitric oxide (NO) was detected using 5 μM 4-amino-5-methylamino-2′,7′-difluorofluorescein diacetate (DAF-FM); and cardiolipin was detected using 80 nM nonyl acridine orange (NAO). All fluorescent probes were loaded at RT for 30 min except TMRM, which was loaded for 10 min to prevent quenching. After loading, cells were rinsed 3 times with HEPES-buffered saline solution (containing 140 mM NaCl, 5.4 mM KCl, 1.8 mM CaCl_2_, 0.8 mM MgCl_2_, 10 mM glucose, 10 mM HEPES; pH 7.4). Cells loaded with the ester form of dyes including DCFH-DA and Rhod-2 required an additional 30–40 min of incubation after dye loading to allow intracellular deacetylation of the dye. Dye-loaded cells were then mounted on a cell chamber for conventional or laser-coupled imaging microscopic observation. 

### Imaging Analysis of Living Cells

Confocal fluorescence images and image stacks were collected using a Zeiss LSM 510 META NLO mounted on an Axiovert 200 M inverted microscope (Carl Zeiss Microimaging, Inc., Thornwood, NY). All fluorescence images were collected using a Zeiss objective lens (Plan-Apochromat 100X, NA1.4 oil DIC M27). NAO was excited using the Argon/2 laser (30 mW) for excitation. The excitation wavelength was 488 nm, the main dichroic beam splitter was 488/561 nm, and the emission detection filter was band pass 500–550 nm.

All images were processed and analyzed using MetaMorph software (Universal Imaging Corp., West Chester, PA, USA). Intensity levels were analyzed from the original images and graphed using Microsoft Excel software and Photoshop. For analyzing mROS and mitochondrial NO (mNO) fluorescent intensity, we selected and measured the regions overlapping with DCFH-DA (to measure ROS) and TMRM (to measure ΔΨm) signals, DAF-FM (to measure NO) and TMRM signals, respectively. Therefore, we were able to make sure that the regions we analyzed were mitochondrial regions. For analyzing fluorescent intensity of cardiolipin, we selected and measured the region overlapping with NAO (to measure cardiolipin) and Rhod-2 (to measure mCa^2+^) to confirm that the sites we measured were mitochondrial regions. To take into account the initial influence of ΔΨm on NAO, we calculated the percentage of fluorescent intensity of NAO signals [100-(The control fluorescent intensity of NAO in mitochondria-the recording fluorescent intensity of NAO in mitochondria) / The control fluorescent intensity of NAO in mitochondria × 100] %. 

### Treatment Protocols of Confocal Experiments on H/RO

To evaluate the effect of hypoxia for 6 h with different reoxygenation durations (1, 2, 3, and 4 h) on different mitochondrial parameters, the cells (n ≥ 6 dishes/group) were treated as follows:

1. Control: cells were stained with different mitochondrial fluorescent dyes for 30 min, and then visualized under a confocal microscope. We double stained cells with 200 nM TMRM and 2 μM DCFH-DA, 200 nM TMRM and 5 μM DAF-FM, or 80 nM NAO and 2 μM Rhod-2 in order to localize the mitochondria and compare different mitochondrial parameter at the same time.
2. Hypoxia for 6 h followed by reoxygenation for 1, 2, 3, and 4 h: cells were subjected to hypoxia for 6 h followed by reoxygenation for 30 min, 1 h 30 min, 2 h 30 min, and 3 h 30 min, respectively. Then cells were stained and visualized as control (1).

To evaluate the effect of different hypoxic durations (6, 12, and 18 h) with the same time of reoxygenation (2h) on cardiolipin depletion and mCa^2+^ accumulation, the cells (n ≥ 6 dishes/group) were treated as follows:

1. Control: cells were double stained with 80 nM NAO and 2 μM Rhod-2 for 30 min, and then visualized at every 3 min for 1 h.
2. Hypoxia for 6, 12, and 18 h followed by reoxygenation for 2 h: cells were subjected to hypoxia for 6, 12, and 18 h; followed by reoxygenation for 1 h 30 min, then stained and visualized as control (1).

To evaluate the effect of H_2_O_2_-augmented H/RO (hypoxia: 6 h; reoxygenation: 2 h) on mROS, cardiolipin, *Δ*Ψm and mCa^2+^ and the protective effects of melatonin treatment, the cells (n ≥ 6 dishes/group) were treated as follows:

1. Control: cells were stained with 2 μM DCFH-DA and 200 nM TMRM, or 80 nM NAO and 2 μM Rhod-2 for 30 min, then 5 mM H_2_O_2_ was added and visualized at every 1 min for 30 min under a confocal microscope.
2. H_2_O_2_ –augmented H/RO treatment (H: 6 h; RO: 2 h): cells were subjected to hypoxia for 6 h and reoxygenation for 1 h and 30 min, then stained and visualized as control (1).
3. Adding 100 μm melatonin during the H_2_O_2_ –augmented H/RO (H: 6 h; RO: 2 h): During the whole procedure of H_2_O_2_ –augmented H/RO treatment, 100 μm melatonin was added to all media and buffers.

### Measurement of Mitochondrial Movement

Mitochondrial movement was continuously time-lapse imaged using a mitochondria-targeted fluorescent probe (either 80 nM cardiolipin or 200 nM TMRM) before and after the cells received H/RO treatment. Mitochondrial movement was measured from the overlapping mitochondrial area (in yellow; i.e., nonmoving area) of a superimposed image of 2 consecutive images (the first image labeled the mitochondrial area red in color and the second image labeled the mitochondrial area green in color) taken 2 min apart. The percentage of the overlapping mitochondrial area of the 2 images (in yellow; i.e., nonmoving area) to total mitochondrial area (counted from the total mitochondrial area of the first image) was designated the overlapping percentage. If the mitochondrial movement became retarded, the overlapping percentage increased (yellow area increased). If the mitochondrial movement accelerated, the overlapping percentage decreased (yellow area decreased). The average of 10 representative populations of mitochondria in one single cell from 10-20 cells was calculated [21].

### Statistical Analysis

Results are expressed as mean ± standard error of the mean (SEM) and statistical significance was evaluated by either one-way or multi-factorial analysis of variance (ANOVA). A P value less than 0.05 was considered statistically significant. Each experiment was repeated at least 3 times.

## Results

### NARP Augments H/RO-induced Apoptosis

**Figure 1:**
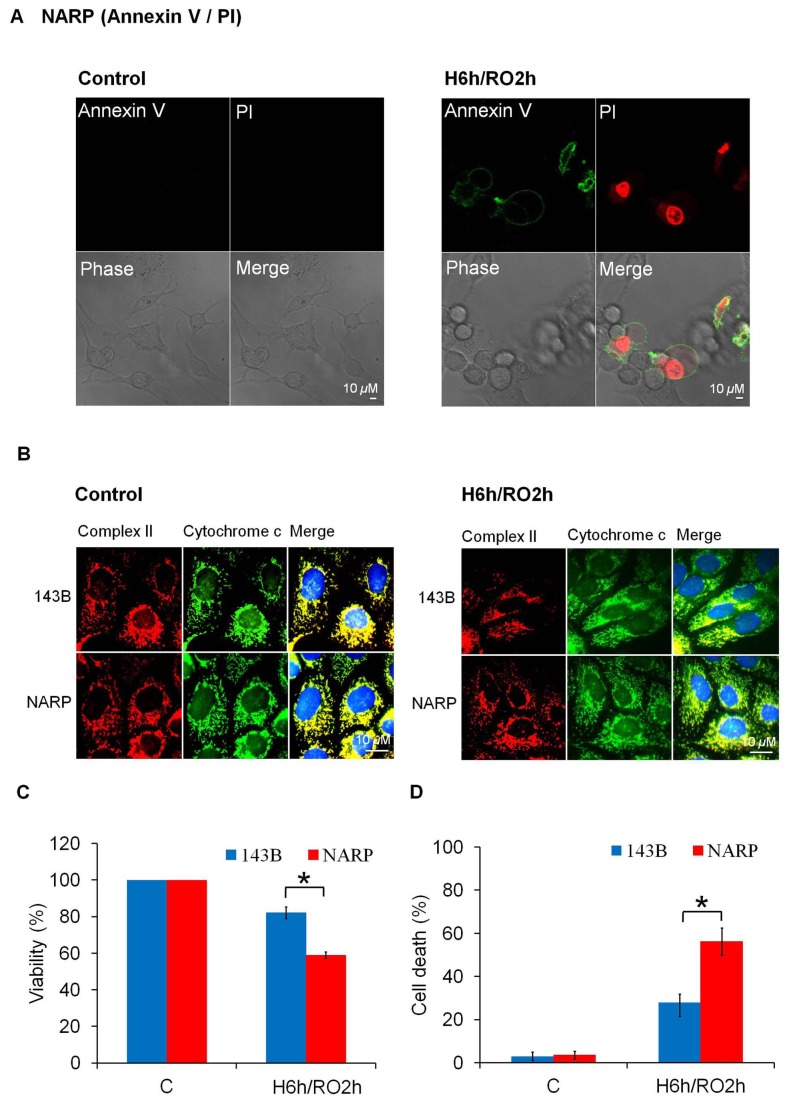
Hypoxia/reoxygenation (H/RO) induced apoptotic death in NARP cybrids and 143B cells. (A) H/RO (H: 6h, RO: 2h) induced apoptosis in NARP cybrids as measured by Annexin V-propidium iodide (PI) staining. (B) H/RO induced cytochrome c release from mitochondria in NARP cybrids and 143B cells as measured by immunocytochemical analysis. Red color: mitochondrial complex II; Green color: cytochrome c. Note that cytochdrome c and complex II co-localized well before H/RO (yellow color). After H/RO, green fluorescent signal was released from mitochondria in NARP cybrids and 143B cells, which suggested that cytochdrome c was released from mitochondria. (C) MTT assay. After H/RO, cell death was noted in NARP cybrids and 143B cells (death ratio: NARP > 143B). (D) Trypan blue exclusion test of cell viability. After H/RO, cell death was noted in NARP cybrids and 143B cells (death ratio: NARP > 143B). *P<0.05 as compared with control; *n*=3.

The effects of NARP-induced inhibition of F1F0-ATPase on cell survival in response to H/RO (H: 6 h; RO: 2 h) stress were detected using Annexin V-PI staining, immuoncytochemical analysis, MTT assays, and the trypan blue exclusion test of cell viability. After H/RO treatment, NARP cybrids showed marked cell death as measured by Annexin V-PI staining (Figure 1A). As shown in Figure 1B, cytochrome c (green fluorescent signal) released from the mitochondria (yellow region) into the cytosol was detected by immunocytochemical analysis in both NARP cybrids and 143B cells in response to H/RO insults. The result of MTT assay demonstrated that NARP-induced inhibition of F1F0-ATPase resulted in more severe apoptotic death in response to H/RO insults as compared with the parental 143B cells (survival rate: 143B [82 ± 3.2%] > NARP [58 ± 2.1%], *P*<0.05) (Figure 1C). The result of the trypan blue exclusion test also showed that NARP-induced inhibition of F1F0-ATPase resulted in more severe cell death in response to H/RO insults as compared with the parental 143B cells (cell death rate: 143B [27 ± 4.1%] < NARP [56 ± 6.3%], *P*<0.05) (Figure 1D). Based on these results, we found that H/RO insults led to apoptotic death in both NARP cybrids and 143B cells. Besides, the NARP-induced inhibition of F1F0-ATPase augmented H/RO-induced apoptosis in comparison to 143B cells.

### NARP Augments H/RO (with the same duration of hypoxia and different durations of reoxygenation)-induced Mitochondrial Dysfunction

**Figure 2:**
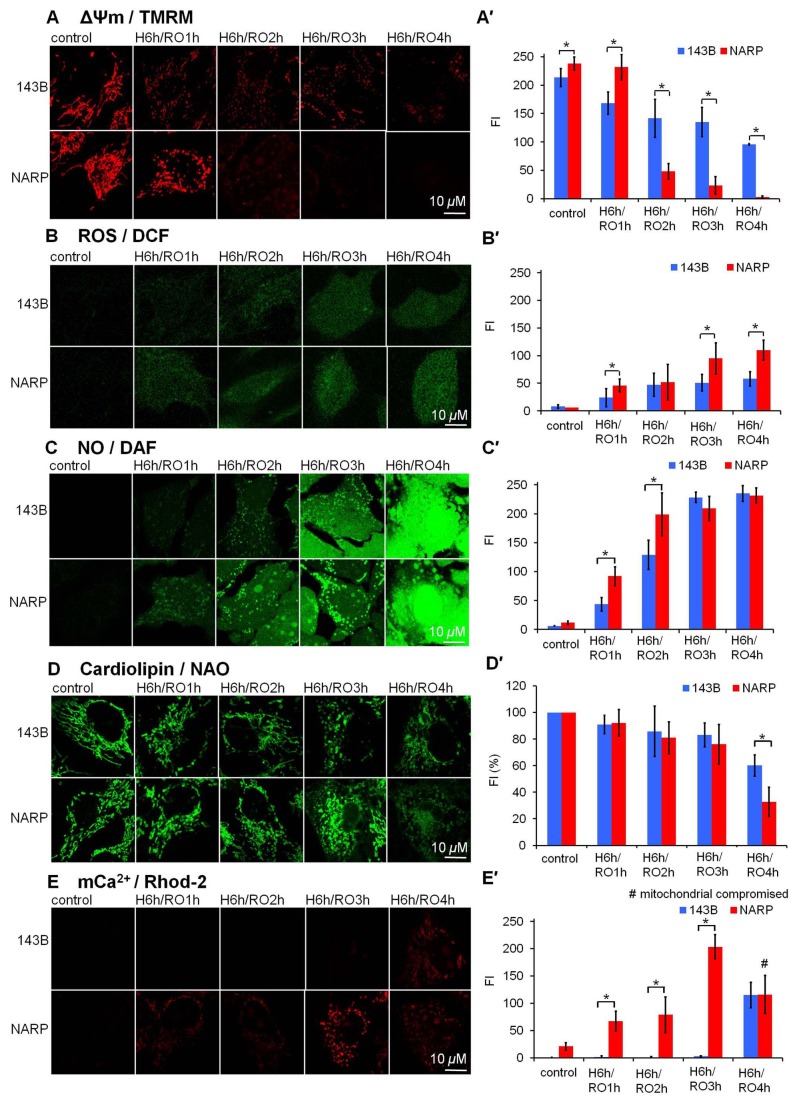
NARP augmented mitochondrial dysfunction upon H/RO. (A) NARP and longer reoxygenation durations augmented H/RO (H: 6 h; RO: 1-4 h)-induced mitochondrial membrane potential (ΔΨm) depolarization. (B) After H/RO with longer reoxygenation duration, mitochondrial reactive oxygen species (mROS) formation increased in both 143B cells and NARP cybrids. NARP augmented mROS formation. (C) After H/RO with longer reoxygenation duration, mitochondrial nitric oxide (mNO) formation increased in both 143B cells and NARP cybrids. NARP augmented the effect of H/RO on mNO formation. (D) Longer reoxygenation durations and NARP augmented cardiolipin depletion. (E) NARP augmented the effect of H/RO on mCa^2+^ accumulation. (A′-E′) Quantitative analyses of A-E. Mitochondrial compromised is indicated (#). P-value representing statistically significant differences between NARP cybrids and 143B cells (*P*<0.05) is indicated (*). n=6.

Next, we investigated how NARP-induced inhibition of F1F0-ATPase augmented H/RO treatment-induced apoptosis. Resting levels of ΔΨm, mROS, mNO, cardiolipin and mCa^2+^ were simultaneously imaged using 200 nM TMRM, 2 μm DCFH-DA, 5 μm DAF-FM, 80 nM NAO and 2 μm Rhod-2, respectively, before and after H/RO treatment in NARP cybrids and 143B cells. We used the same duration of hypoxia (6 h) and different durations of reoxygenation (1, 2, 3, and 4 h) to simulate dose-dependent H/RO insults. As shown in Figure 2A and 2A’, NARP resulted in hyperpolarized ΔΨm as compared with the parental 143B cells before H/RO treatment (*P*< 0.05), which was similar to what we observed in our previous study [21]. The ΔΨm in NARP cybrids, although more hyperpolarized before H/RO treatment, were depolarized much more severely than ΔΨm in 143B cells after H/RO treatment with longer reoxygenation durations (2-4 h) (ΔΨm of NARP < ΔΨm of 143B when reoxygenation durations were 2-4 h, *P*<0.05), suggesting NARP-induced hyperpolarization of ΔΨm was vulnerable to the H/RO insult, which was similar to what we noted in our previous study with other apoptotic insults [21]. Figure 2B and 2B’ showed that NARP augmented dose-dependent H/RO insults-induced mROS formation (mROS of NARP > mROS of 143B when reoxygenation duration was 3-4 h, *P*<0.05). In NARP cybrids, higher levels of mROS associated with more severe depolarization of ΔΨm suggested that NARP-induced inhibition of F1F0-ATPase augmented mROS (induced by H/RO insult)-induced ΔΨm depolarization. 

Furthermore, we investigated how H/RO insults-induced mROS formation altered cardiolipin content, a critical mitochondrial protective phosphopholipid and the levels of mCa^2+^, another potent mitochondrial stress that activates the opening of mPTP. Resting levels of cardiolipin and mCa^2+^ were simultaneously imaged using 80 nM NAO and 2 μm rhod-2, respectively, before and after H/RO treatment (H: 6 h; RO: 1-4 h) in NARP cybrids and 143B cells. Compared with 143B cells, the resting level of mCa^2+^ in NARP cybrids was higher, which was due possibly to the inhibition of F1F0-ATPase-induced hyperpolarization of ΔΨm (*P*<0.05) (Figure 2E and 2E’). After H/RO treatment, dose dependency of H/RO insult-induced mCa^2+^ accumulation was noted in both 143 B cells and NARP cybrids, NARP augmented this effect significantly (mCa^2+^ of NARP > mCa^2+^ of 143B when reoxygenation duration was 1-3 h, *P*<0.05) (Figure 2E and 2E’). Figure 2D and 2D’ showed that NARP-induced inhibition of F1F0-ATPase augmented the depletion of cardiolipin in response to H/RO insult (RO: 4h) (>50%, *P*<0.05). Interestingly, the event of cardiolipin depletion happened later (longer duration of reoxygenation) than ΔΨm depolarization, mROS formation and mCa^2+^ accumulation, suggesting that NARP-induced inhibition of F1F0-ATPase augmented mROS (induced by H/RO insult)-induced ΔΨm depolarization and mCa^2+^ accumulation, which resulted in the depletion of cardiolipin, the opening of mPTP, and eventually cell death. We also observed that NARP augmented mNO formation as compared with 143 B cells in response to dose-dependent H/RO treatment (Figure 2C and 2C’), which may also contribute to H/RO insult-induced cell death.

### NARP Augments Longer Duration of Hypoxia (with the Same Duration of Reoxygenation)-induced Cardiolipin Depletion and mCa^2+^ Accumulation

**Figure 3:**
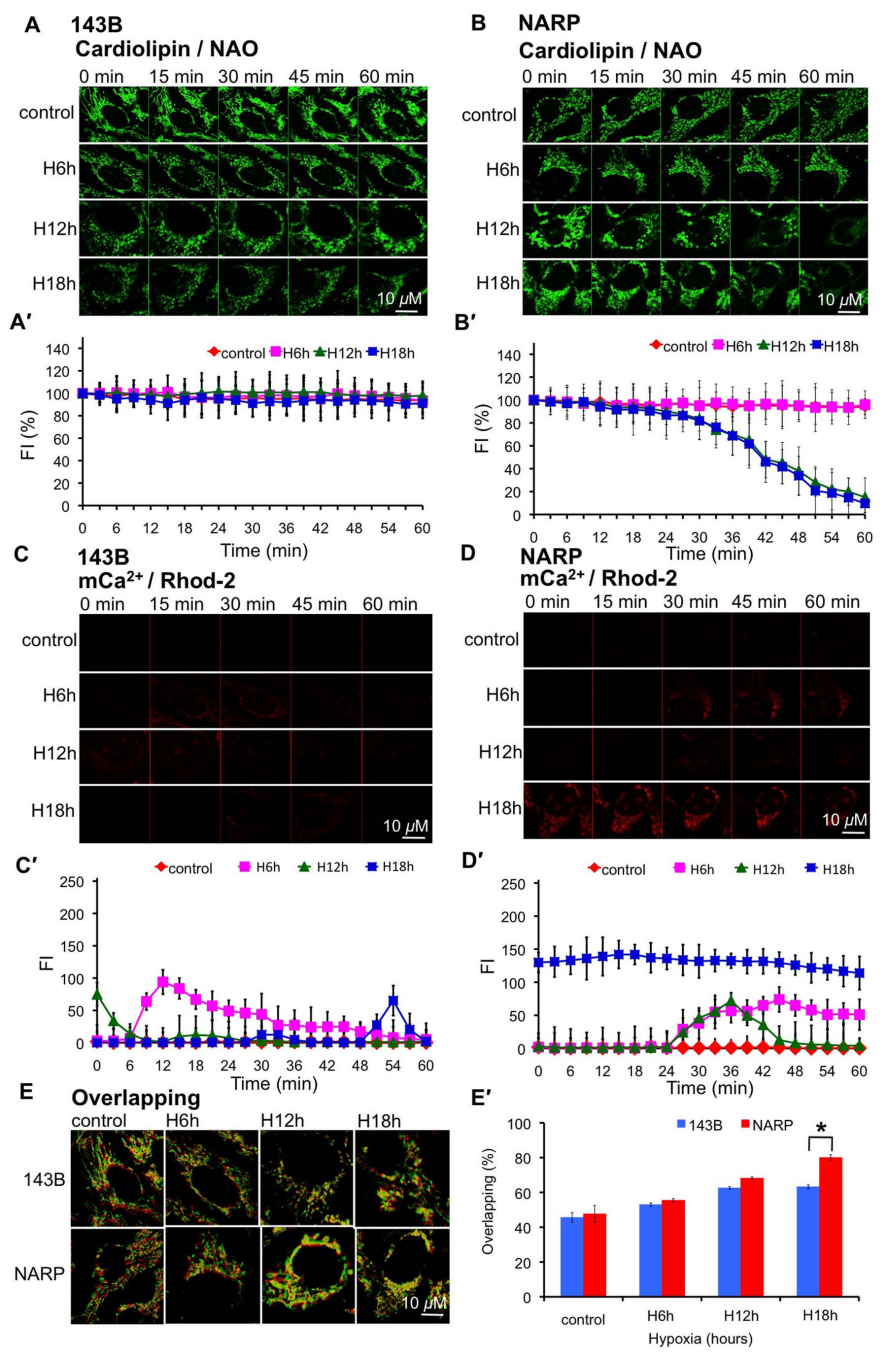
Effects of H/RO (with different hypoxia durations) on mitochondrial functions in NARP cybrids and 143B cells. (A) After treating 143B cells with H/RO (H: 6, 12, and 18 h; RO: 2 h), no obvious cardiolipin depletion was noted during recording. Mitochondria remained thread-like. (B) Cardiolipin depletion was noted gradually in NARP cybrids receiving H/RO with durations of hypoxia for 12 and 18 h. Mitochondria became swollen and round. (C) In 143B cells, only transient mCa^2+^ accumulation was noted (D) Marked and persistent mCa^2+^ accumulation was noted in NARP cybrids exposed to H/RO with duration of hypoxia for 18 h. (E) Red/green overlay of two consecutive confocal images (Δt = 2min) of 80 nM nonyl acridine orange (NAO) fluorescence in NARP cybrids and 143B cells upon H/RO with different hypoxia durations. The percentage of nonmoving mitochondria (overlapping mitochondrial area, in yellow color) to total mitochondrial area increased from 45 ± 3.2% to 63 ± 1.4% in 143B cells and 48 ± 5.1% to 77 ± 2.3% in NARP cybrids after the exposure of cells to H/RO with duration of hypoxia for 18 h. P-value representing statistically significant differences between NARP cybrids and 143B cells is indicated (*). (A′-E′) Quantitative analyses of A-E. n=6.

Due to the depletion of cardiolipin occurring later than mROS formation, ΔΨm depolarization and mCa^2+^ accumulation in response to H/RO treatment, we next investigated the effects of longer duration of hypoxia (with the same duration of reoxygenation) on cardiolipin content in NARP cybrids and 143B cells. After H/RO treatment (H: 0, 6, 12, and 18 h; RO: 2 h), cells were stained with 80 nM NAO (to measure cardiolipin) and 2 μm Rhod-2 (to measure mCa^2+^). Then the cell images were recorded by confocal microscopy at every 3 min to monitor the resting change of cardiolipin content and mCa^2+^. Interestingly, depletion of cardiolipin was noted gradually after H/RO treatment (H: 12 and 18 h; RO: 2h) in NARP cybrids (*P*<0.05, at 42 min and 60 min) but not in 143B cells (Figure S1A-B; Figure 3A, A′, B, B′). In addition, marked and persistent accumulation of mCa^2+^ was noted after H/RO treatment in NARP cybrids but not in 143B cells exposed to hypoxia for 18 h (*P*<0.05, H1h group compared with control group) (Figure S1D; Figure 3D, D′). Only a small amount and transient accumulation of mCa^2+^ was noted in 143B cells (Figure S1C; Figure 3C, C′). Furthermore, the mitochondrial morphology of 143B cells remained thread-like when receiving H/RO treatment with hypoxia for 12 and 18 h (Figure 3A). However, the mitochondrial morphology of NARP cybrids was characterized by swelling and roundness when receiving H/RO treatment with hypoxia for 12 and 18 h (Figure 3B). These results, thus, indicate that the NARP cybrids were more sensitive to longer hypoxic duration (with the same duration of reoxygenation)-induced cardiolipin depletion and of mCa^2+^ accumulation. Our previous study had suggested that cardiolipin possibly plays a central role in regulating mitochondrial dynamics that is associated with NARP-augmented pathology and is crucial for maintaining normal mitochondrial movement [21]. Therefore, we proposed that the NARP-enhanced depletion of cardiolipin in response to H/RO insults may lead to more severe retardation of mitochondrial movement in comparison to 143B cells.

### NARP Augments H/RO-induced Retardation of Mitochondrial Movement

We next investigated whether NARP augmented the retardation of mitochondrial movement in response to H/RO treatment (H: 0, 6, 12, and 18 h; RO: 2 h). We loaded the cells with 80 nM NAO, and mitochondrial movement was analyzed using time-lapse imaging continuously in 143B cells and NARP cybrids. Before H/RO treatment, the NARP cybrids did not show significantly reduced mitochondrial movement compared with the 143B cells, so that the non-moving mitochondrial population analyzed from the percentage of the overlapping area (yellow area) of two consecutive images (the first image labeled red in color and the second image labeled green in color, taken 2 min apart) to total mitochondrial area (mitochondrial area in the first image) was 45 ± 3.2% in 143B and 48 ± 5.1% in NARP cells. After H/RO treatment (with different durations of hypoxia and the same duration of reoxygenation), the non-moving mitochondrial population analyzed from the percentage of the overlapping area (yellow area) of two consecutive images to total mitochondrial area increased to 63 ± 1.4% in 143B cells and to 77 ± 2.3% in NARP cybrids after the exposure of cells to hypoxia for 18 h (Figure 3E, E′). Interestingly, the percentage of the ratio of nonmoving mitochondria to the entire mitochondrial population after and before H/RO treatment (H: 18 h; RO: 2 h) was higher in NARP cybrids (NARP: 160% and 143B: 140%, *P*<0.05). These results suggested that the NARP-induced inhibition of F1F0-ATPase significantly augmented the retardation of mitochondrial movement after H/RO treatment with prolonged duration of hypoxia.

### Melatonin Reduces H/RO-induced Apoptosis in Both NARP cybrids and 143B Cells

**Figure 4:**
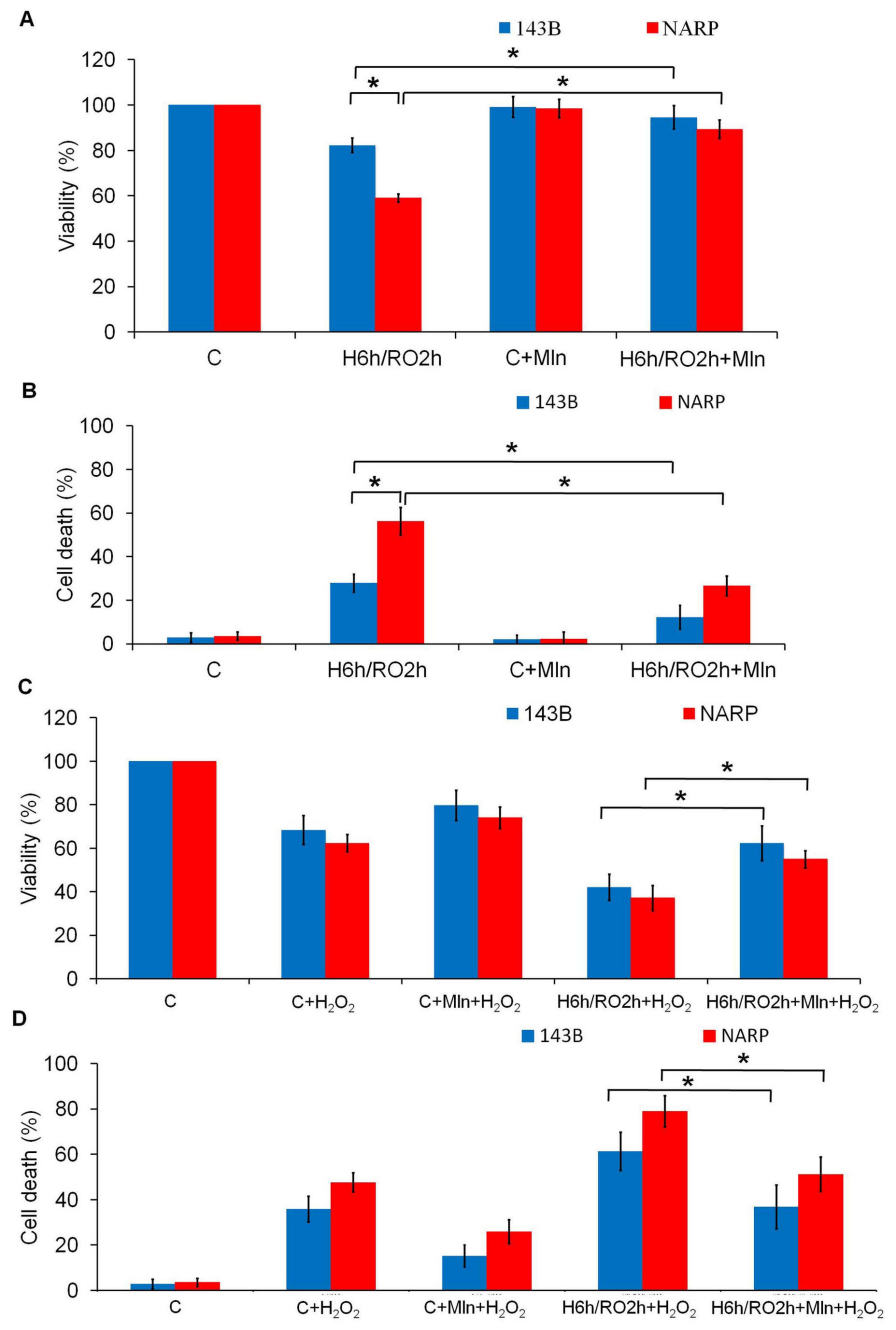
Effects of melatonin on cell viability in NARP cybrids and 143B cells in response to H/RO. (A) MTT assay. After H/RO (H: 6h, RO: 2h), apoptotic death was noted in NARP cybrids and 143B cells (death ratio: NARP > 143B) (column 2). Adding melatonin 100μm during H/RO significantly reduced NARP-augmented cell death (column 4). (B) Trypan blue exclusion test of cell viability. Addition of 100 μm melatonin during H/RO treatment reduced the percentage of cell death in both 143B and NARP cells (columns 2 and 4). (C) MTT assay. Administering melatonin 100μm during H_2_O_2_ 5 mM-augmented H/RO improved cell survival in both NARP cybrids and 143B cells (column 4 and 5). (D) Trypan blue exclusion test of cell viability. Addition of melatonin during H/RO treatment reduced the percentage of cell death in 143B and NARP cybrids in response to H_2_O_2_-augmented H/RO insults (columns 4 and 5). Each value represents the mean ± S.E. of three independent determinations. P-values representing statistically significant differences between NARP and 143B cells upon H/RO; without melatonin and with melatonin during H/RO (both in the groups with and without secondary H_2_O_2_ stress) are indicated (*). Mln: melatonin.

Next, we investigated that whether melatonin, a potent mitochondrial protector, has a protective effect in response to H/RO insults in NARP cybrids and 143B cells, We used H/RO treatment with hypoxia for 6 h and reoxygenation for 2 h in the following experiments since it could have induced moderate effects on mitochondrial parameters in previous dose-dependent experiments. The result of MTT assay demonstrated that addition of 100 μm melatonin during H/RO (H: 6 h, RO: 2 h) treatment improved the percentage of cell survival in both 143B and NARP cells from 82 ± 3.2% and 58 ± 2.1% to 95 ± 5.1% and 89 ± 4.3%, respectively (*P*<0.05) (Figure 4A columns 2 and 4). The trypan blue exclusion test of cell viability also showed that addition of 100 μm melatonin during H/RO (H: 6 h, RO: 2 h) treatment reduced the percentage of cell death in both 143B and NARP cells from 27 ± 4.1% and 56 ± 6.3% to 12 ± 5.4% and 27 ± 4.6%, respectively (*P*<0.05) (Figure 4B columns 2 and 4). For generating a stress mimic of stronger reoxygenation insults, we added a secondary oxidative stress (H_2_O_2_ 5 mM) following H/RO treatment to augment the H/RO insults. The result of MTT assay demonstrated that addition of melatonin during H/RO treatment improved cell survival in 143B cells and NARP cybrids in response to H_2_O_2_-augmented H/RO insults from 42 ± 6.1% and 37 ± 6.3% to 62 ± 8.1% and 55 ± 4.2%, respectively (*P*<0.05) (Figure 4B. columns 4 and 5). The trypan blue exclusion test of cell viability also showed that addition of melatonin during H/RO treatment reduced the percentage of cell death in 143B cells and NARP cybrids in response to H_2_O_2_-augmented H/RO insults from from 61 ± 8.5% and 79 ± 6.9% to 37± 9.6% and 51 ± 7.5%, respectively (*P*<0.05) (Figure 4B. columns 4 and 5)

### Melatonin Reduces NARP-enhanced H/RO-induced mROS Formation

**Figure 5:**
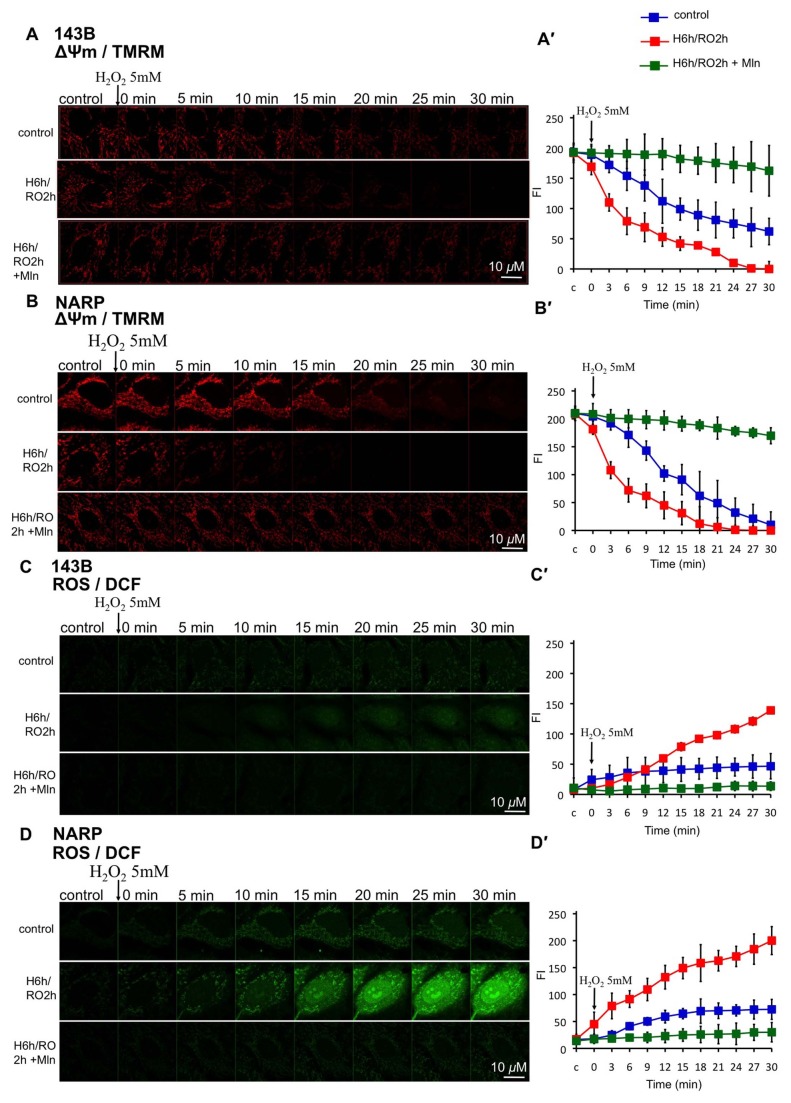
Effects of melatonin on ΔΨm and mROS upon H_2_O_2_-augmented H/RO in 143B cells and NARP cybrids. (A, B) In both 143B cells and NARP cybrids, ΔΨm depolarization was faster in the group with H_2_O_2_ (5mM)-augmented H/RO (H: 6 h; RO: 2 h); NARP augmented this effect. Adding melatonin 100 μm during H_2_O_2_-augmented H/RO effectively protected ΔΨm from depolarization. (C) In 143B cells, more mROS formation was noted in the group of H_2_O_2_-augmented H/RO. Adding melatonin during H_2_O_2_-augmented H/RO effectively suppressed mROS formation. (D) In NARP cybrids, mROS formation was augmented as compared with 143B cells. Adding melatonin during H_2_O_2_-augmented H/RO still effectively suppressed mROS formation. (A′-D′) Quantitative analyses of A-D. n=6. FI: fluorescence intensity. Mln : melatonin.

To explore whether melatonin-reduced apoptotic death in response to H/RO insult is through suppressing mROS formation and protecting ΔΨm, and whether NARP-induced inhibition of F1F0-ATPase disrupt these effects, we measured mROS using 2 μm DCFH-DA and 200 nM ΔΨm using TMRM in NARP cybrids and 143B cells after H/RO treatment (H: 6h, RO: 2h) and administering H_2_O_2_ 5mM following H/RO treatment. It was obvious that H_2_O_2_-augmented H/RO insults induced a significant mROS formation in both NARP cybrids and 143B cells. Moreover, NARP greatly enhanced the effect of mROS formation (fluorescent intensity of DCF, recorded at 30 min after adding H_2_O_2_ NARP/143B=1.5, *P*<0.05)(Figure S2C-D; Figure 5C, C′, D, D′). Adding 100 μm melatonin during H_2_O_2_-augmented H/RO treatment suppressed mROS formation and protected ΔΨm from depolarization in NARP cybrids and 143B cells (*P*<0.05) (Figure S2, Figure 5). The above results indicated that the protection of NARP cybrids and 143B cells from H_2_O_2_-augmented H/RO treatment by melatonin was due to suppression of mROS formation and ΔΨm depolarization. Moreover, the NARP-induced inhibition of F1F0-ATPase augmented mROS formation significantly in response to H_2_O_2_-augmented H/RO treatment, but melatonin still could suppress mROS formation in NARP cybrids.

### Melatonin Reduces mROS-mediated Cardiolipin Depletion and mCa^2+^ Accumulation upon H/RO in Both NARP Cybrids and 143B Cells

**Figure 6:**
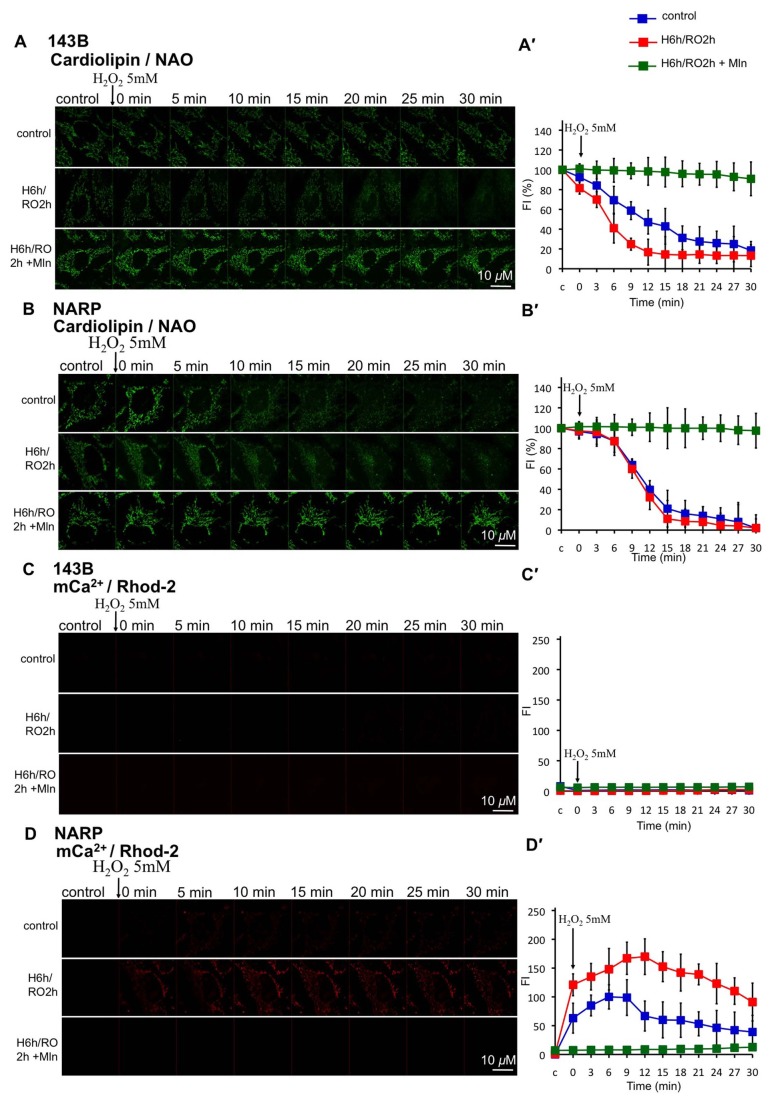
Effects of Melatonin on cardiolipin and mCa^2+^ upon H_2_O_2_ –augmented H/RO in 143B cells and NARP cybrids. (A) In 143B cells, cardiolipin depletion was augmented in the group with H_2_O_2_-augmented H/RO (H: 6 h; RO: 2 h). Note that adding melatonin 100 μm during H_2_O_2_-augmented H/RO protected cardiolipin from depletion. (B) In NARP cybrids, cardiolipin depletion was augmented as compared with 143B cells, and adding melatonin during H_2_O_2_-augmented H/RO protected cardiolipin from depletion. (C) In 143B cells, no obvious mCa^2+^ accumulation was noted in response to H_2_O_2_-augmented H/RO. (D) In NARP cybrids, mCa^2+^ accumulation was augmented in the group with H_2_O_2_-augmented H/RO. Adding melatonin during H_2_O_2_-augmented H/RO suppressed mCa^2+^accumulation. (A′-D′) Quantitative analyses of A-D. n=6. Mln: melatonin.

To investigate how melatonin inhibited NARP-enhanced mROS formation in response to H_2_O_2_-augmented H/RO insults, and if the content of cardiolipin and the levels of mCa^2+^ were altered, we measured cardiolipin using 80 nM NAO and mCa^2+^using 2 μm Rhod-2 at the same time after H/RO treatment. After H_2_O_2_-enhanced H/RO insults, NARP-induced inhibition of F1F0-ATPase significantly augmented the mROS-mediated depletion of cardiolipin in comparison with 143B cells (NAO fluorescent intensity (%) in 143B/ NAO fluorescent intensity (%) in NARP = 7, recording at 30min after adding H_2_O_2_, P<0.05) (Figure S3A-B; Figure 6A, A′, B, B′). NARP also enhanced the accumulation of mCa^2+^ in response to H_2_O_2_-enhanced H/RO insults (*P*<0.05, compared between H/RO group of NARP and 143B cells). No obvious mCa^2+^ accumulation was noted in 143B cells upon H/RO treatment (fluorescence intensity <10 during recording) (Figure S3C-D; Figure 6C, C′, D, D′). Interestingly, melatonin (100 μm) significantly protected mROS-mediated depletion of cardiolipin in both NARP cybrids and 143B cells upon H_2_O_2_-augmented H/RO treatment (*P*<0.05) (Figure S3A-B; Figure 6A, A′, B, B′). In addition, melatonin also suppressed mCa^2+^ accumulation in response to H_2_O_2_-augmented H/RO insults in NARP cybrids (*P*<0.05) (Figure S3C-D; Figure 6C, C′, D, D′). These results indicated that the NARP-induced inhibition of F1F0-ATPase greatly enhanced mROS formation and mCa^2+^ accumulation after H_2_O_2_-augmented H/RO insults, which led to mROS-mediated depolarization of ΔΨm and depletion of cardiolipin. Melatonin significantly prevented mROS-mediated depletion of cardiolipin upon H_2_O_2_-augmented H/RO treatment in both NARP cybrids and 143B cells. To investigate if the melatonin-related protection is primary through reducing overall mROS, we administered a mitochondrial specific antioxidant mitoQ (0.2 nM) or a general antioxidant vitamin E (200 μm) during H_2_O_2_-augmented H/RO insults for comparison and evaluated their protective effects on mROS, ΔΨm, mCa^2+^, and cardiolipin (Figure S4-7). The effect of mitoQ on mROS suppression was better than vitamin E in response to H_2_O_2_-augmented H/RO insults (Figure S4, S6). 

### Melatonin Improves H/RO-induced Retardation of Mitochondrial Movement in Both NARP Cybrids and 143B Cells

**Figure 7:**
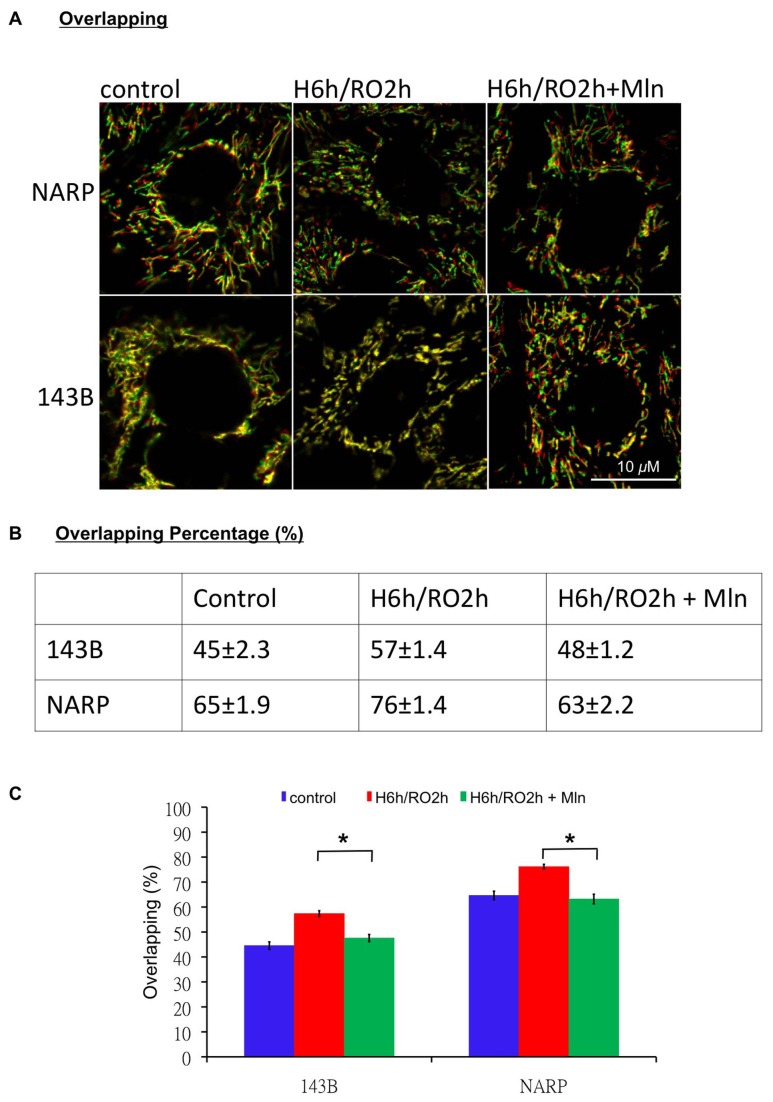
Melatonin significantly improved H/RO-induced retardation of mitochondrial movement in 143B cells and NARP cybrids. (A, B) Red/green overlay of two consecutive confocal images (Δt = 2min) of 200 nM tetramethylrhodamine methyl ester (TMRM) fluorescence in NARP cybrids and 143B cells in response to H/RO treatment without and with 100 μm melatonin. H/RO (H: 6 h; RO: 2 h) reduced the velocity of mitochondrial movement in both 143B cells and NARP cybrids, so that the percentage of nonmoving mitochondria (overlapping mitochondrial area, in yellow color) to the entire mitochondria were 57 ± 1.4% in 143B cells and 76 ± 1.4% in NARP cybrids as compared to the cells and cybrids, respectively, without H/RO treatment. Adding melatonin 100 μm during H/RO improved mitochondrial movement in both 143B cells and NARP cybrids as compared with the groups without melatonin during H/RO, so that the percentage of nonmoving mitochondria to the all mitochondria was 48 ± 1.2% in 143B cells and 63 ± 2.2% in NARP cybrids. (C) Quantitative analyses of (A). *P<0.05. n=6. Mln: melatonin.

Finally, we investigated whether the melatonin-induced protection of cardiolipin in NARP cybrids and 143B cells improved the retardation of mitochondrial movement in response to H/RO insults. After H/RO treatment (H: 6h, RO: 2h), we loaded the cells with 200 nM TMRM, and mitochondrial movement was analyzed using time-lapse imaging continuously both with and without melatonin in 143B cells and NARP cybrids. H/RO treatment significantly induced the retardation of mitochondrial movement in 143B cells and NARP cybrids, so that the non-moving mitochondrial population analyzed from the percentage of the overlapping area (yellow area) of two consecutive images (the first image labeled red in color and the second image labeled green in color, taken 2 min apart) to total mitochondrial area (mitochondrial area in the first image) increased from 45 ± 2.3% to 57 ± 1.4% in 143B cells (*P*<0.05), and from 65 ± 1.9% to 76 ± 1.4% in NARP cybrids (*P*<0.05). Administering 100 μm melatonin during H/RO treatment significantly improved the retardation of mitochondrial movement, so that the non-moving mitochondrial population analyzed from the percentage of the overlapping area (yellow area) of two consecutive images to total mitochondrial area decreased from 57 ± 1.4% to 48 ± 1.2% in 143B cells (*P*<0.05), and from 76 ± 1.4% to 63 ± 2.2% in NARP cybrids (*P*<0.05) (Figure 7). 

## Discussion

**Figure 8:**
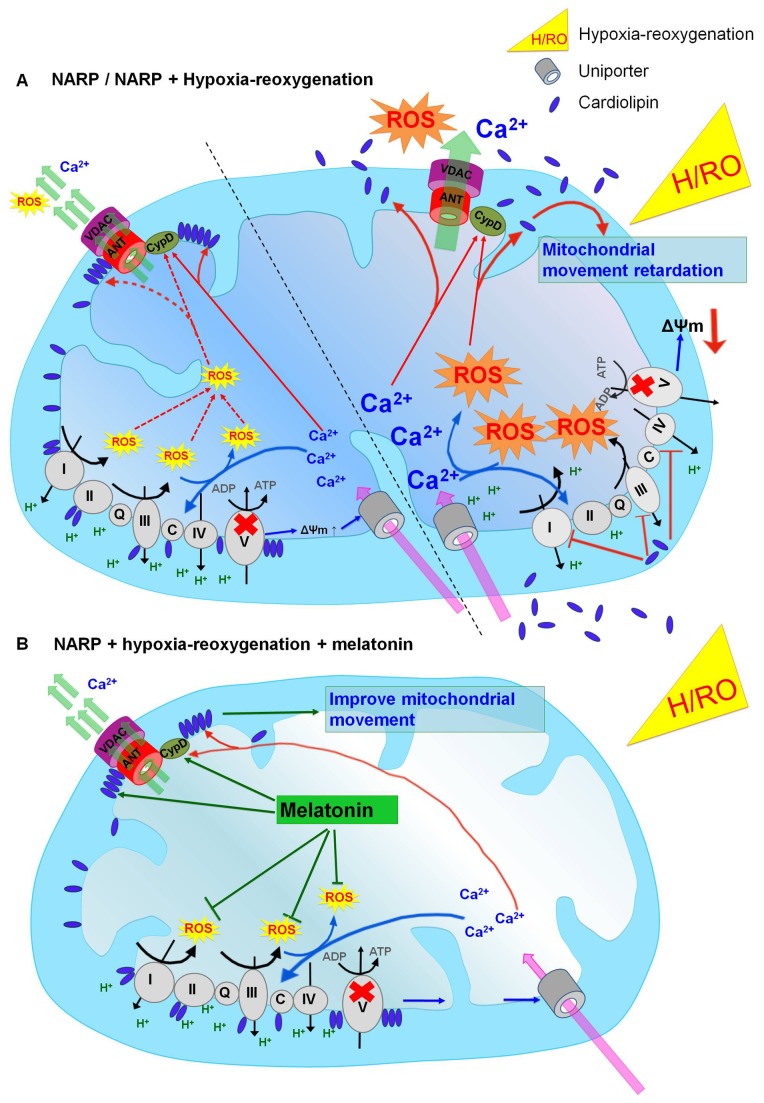
Schematic illustration of NARP-induced F1F0-ATPase defect **augments mitochondrial dysfunction upon H/RO**. (A) Control NARP cybrids. The NARP mutation-induced inhibition of F1F0-ATP synthase results in hyperpolarization of the ΔΨm, which leads to enhanced basal mROS formation and mCa^2+^ accumulation. Both mROS and mCa^2+^ act synergistically to deplete cardiolipin, which leads to inadequate protective function of transient mitochondrial permeability transition (MPT) as compared with wild-type cells (left hand side). With H/RO, F1F0-ATP synthase fails to work in the reverse mode in NARP cybrids due to reduced ATP hydrolysis and unhealthy enzyme function, which results in depolarization of ΔΨm. In addition, NARP mutation augments H/RO-induced mROS formation and mCa^2+^ accumulation, which leads to more severe depletion of cardiolipin. The depletion of cardiolipin results in mitochondrial movement retardation, inhibition of complexes I, III and IV of the respiratory chain, and fatal opening of MPT pore (right hand side). (B) In the presence of melatonin during H/RO, H/RO-induced mROS formation is greatly reduced, which stabilize cardiolipin. Cardiolipin stabilization results in preservation of ΔΨm, improvement of mitochondrial movement, and suppressing fatal MPT.

In the present study, by using the cells with NARP-induced inhibition of F1F0-ATPase, we demonstrated for the first time that NARP-induced inhibition of F1F0-ATPase-augmented H/RO insult-induced apoptosis. NARP-augmented H/RO insult was closely associated with a pathological enhancement of mROS formation, which led to accumulation of mCa^2+^, depolarization of ΔΨm, and more severe depletion of the protective mitochondrial phospholipid cardiolipin. The above results led to the more severe retardation of mitochondrial movement, and then activated the opening of mPTP in NARP cybrids. Interestingly, melatonin significantly improved cells survival by preventing mROS-mediated cardiolipin depletion and mCa^2+^ accumulation, and rescued the retardation of mitochondrial movement in NARP cybrids in response to H/RO insults. To our knowledge, this is the first report to elucidate the influence of NARP-induced inhibition of F1F0-ATPase on H/RO insults-induced mitochondrial dysfunction and the protective action of melatonin in NARP cybrids in response to the H/RO insults. The precise schematic illustration of NARP-induced inhibition of F1F0-ATPase augmentation of mitochondrial dysfunction upon H/RO treatment and the protections by melatonin in NARP cybrids is shown in Figure 8.

The reason that NARP-induced F1F0-ATPase inhibition augments mROS formation in response to H/RO treatment is still unknown. Previous studies suggested that the hyperpolarized ΔΨm in NARP cells leads to the decreased activity of the mitochondrial respiratory chain as a consequence of F1F0-ATPase inhibition and mitochondrial coupling thus resulting in enhanced mROS formation [34,35]. Our recent study had also demonstrated that NARP enhanced mROS formation upon other apoptotic insults (e.g., amyloid-βtreatment and focal laser irradiation-induced ROS stress) [36]. In addition, our group also previously observed that defect of mtDNA augmented mROS formation with enhancement of apoptosis in common deletion (mtDNA 4977 bp deleted) cybrids [37,38] and in RBA-1 astrocytes containing defective mitochondrial complex I because of long-term rotenone exposure (RT-RBA-1 cells, unpublished data) suggesting that mtDNA mutations or complex defects may potentially enhance several neurodegenerative disorders and even the H/RO injury. 

Our group previously demonstrated that cardiolipin is a crucial pathological target for mitochondrial apoptotic insults (e.g., H_2_O_2_, arachidonic acid, ionomycin) in NARP cybrids, [21]. In the current study, we demonstrated that cardiolipin is an important pathological target for H/RO insults in cells with NARP-induced inhibition of F1F0-ATPase. Previous studies had demonstrated that NARP enhances the production of toxic mROS [35,39]. In this study, we confirmed that NARP-augmented mROS formation led to more severe depletion or peroxidation of cardiolipin. It is well known that mROS-induced cardiolipin peroxidation leads to impaired mitochondrial function and depressed respiratory chain [40-42]. mROS production, cardiolipin depletion/peroxidation, and respiratory chain impairment are linked to each other to create a vicious cycle that leads to the decline of mitochondrial bioenergetics and subsequent mitochondrial dysfunction associated with H/RO insults [43]. In addition, peroxidized cardiolipin can behave as an inducer of mPTP opening, which lowers the threshold of Ca^2+^ for inducing this process and/or potentiating the effect of Ca^2+^ in mPTP opening. The effect of peroxidized cardiolipin on mPTP is associated with a release of cytochrome *c* from the mitochondria [44,45]. It is thus conceivable that NARP-augmented mROS formation upon H/RO treatment induces more severe depletion and peroxidation of cardiolipin, which contribute to mPTP opening, cytochrome c release, and more severe cell death. 

NARP-induced inhibition of F1F0-ATPase possibly augments the retardation of mitochondrial movement in response to H/RO insults through the depletion of cardiolipin. Our previous study suggested that cardiolipin is a crucial modulator for the interaction between mitochondria and motor proteins of the microtubule for maintaining normal mitochondrial movement [21]. Upon mitochondrial apoptotic insults (we used H_2_O_2_, arachidonic acid or ionomycin treatment), mitochondria lose cardiolipin, which may result in retardation of mitochondrial movements. We propose that NARP-augmented depletion of cardiolipin upon H/RO insults leads to weakening the mitochondria-microtubule interaction by loss of cardiolipin in mitochondria, and results in retardation of mitochondrial movement.

The beneficial effects of melatonin (N-acetyl-5-methoxy-tryptamine) on human health are well known and are frequently associated with its antioxidant, or free radical-scavenging activity. The physiological distribution of melatonin is highest in the cell membrane, followed by mitochondria, nucleus, and cytosol [46]. The most unique property of melatonin is that its metabolites also have the ability to scavenge ROS and reactive nitrogen species. The continuous protection exerted by melatonin and its metabolites, referred to as the free radical scavenging cascade, makes melatonin highly effective, even at low concentrations, for protecting organisms from oxidative stress [47-49]. *N* ^1^-acetyl-*N* ^2^-formyl-5-methoxykynuramine (AFMK) and *N*[1]-acetyl-5-methoxykynuramine (AMK), the metabolites of melatonin, have been found to exhibit protective effects against oxidative stress. In general, their protective activities against oxidative stress follow the order AMK > melatonin > AFMK. The efficiency of melatonin for scavenging free radicals is predicted to be reduced when it is metabolized to AFMK and the efficiency of melatonin for scavenging the radicals in aqueous solution is predicted to be increased when it is metabolized to AMK [50,51]. The direct free radical scavenging activity of melatonin has been extensively studied. Interestingly, melatonin also has indirect antioxidative effects via the stimulation of antioxidative enzymes [52]. However, our results did not differentiate between the direct and indirect antioxidative effects of melatonin in response to H/RO treatment. Furthermore, in addition to being a broad-spectrum antioxidant, melatonin is a ligand of several G-protein-coupled receptors. Two mammalian isoforms of the melatonin receptor (melatonin receptor 1 and 2) were identified in a previous study [53]. Previous studies have suggested that the melatonin receptor–ligand axis may play a pathogenic role in several neurodegenerative diseases and is critical for neuroprotection. Therefore, the indirect antioxidative effects of melatonin are presumed to be receptor-mediated [54].

Intriguingly, our result demonstrated that melatonin could preserve cardiolipin, prevent ΔΨm depolarization, suppress ROS formation, prevent mCa^2+^ accumulation, and rescue retardation of mitochondrial movement upon H/RO insults in both NARP cybrids and 143B cells. Moreover, NARP-induced inhibition of F1F0-ATPase did not disrupt the protection generated by melatonin in response to H/RO insults. Several recently published studies showed that melatonin and several of its metabolites (e.g., AFMK, AMK) have significant protective actions against cardiac damage induced during H/RO treatment [23-27,55]. The possible mechanisms of the protective effect of melatonin during H/RO treatment include: (1) melatonin is a potent and broad-spectrum antioxidant that antagonizes mitochondrial oxidative stress (2), melatonin can preserve the content and integrity of cardiolipin molecules, which inhibit mPTP opening through cardiolipin protection [56,57], (3) melatonin inhibits the opening of mPTP directly and this contributes to its neuroprotective effect in cerebral ischemia [56], (4) melatonin may have a direct targeting effect on mCa^2+^-mediated apoptotic events. Peng et al. recently suggested that melatonin directly stabilizes cardiolipin to prevent its depletion and peroxidation, which leads to improvement of mitochondrial movement [21]. Our finding in this study demonstrating that melatonin can rescue retardation of mitochondrial movement in NARP cybrids and 143B cells upon H/RO stress was in agreement with Peng et al’s study. The reason that NARP-induced inhibition of F1F0-ATPase does not disrupt the mitochondrial protective effects of melatonin upon H/RO treatment is still being studied. Mattiazzi et al. had previously showed that antioxidants restore respiration and partially rescue ATP synthesis in NARP cybrids, suggesting that free radical-mediated inhibition of OXPHOS contributes to the loss of ATP synthesis [39]. Theoretically, the function of the electron transport chain (complex I to IV) is intact in NARP cybrids (which only have complex V defect), therefore, melatonin may also act on the electron transport chain but not on F1F0-ATPase to generate its mitochondrial protective effect upon H/RO treatment. Comparing the differences between NARP cybrids and 143B cells during H/RO treatment in our data, it was obvious that NARP-induced inhibition of F1F0-ATPase augmented mCa^2+^ accumulation during H/RO treatment, either by enhancement with longer reoxygenation duration, longer hypoxia duration or secondary oxidative stress. Further experiments such as using Ca^2+^-free HEPES solution or treating NARP cells with Ruthenium red to inhibit mCa^2+^uniporter upon H/RO insults may resolve this problem. Furthermore, previous studies suggest that melatonin could maintain an optimal ΔΨm by regulating the mPTP [56,58]. Under normal conditions, melatonin activates the mPTP and mildly reduces the ΔΨm. This process is associated with mitochondrial oxidative phosphorylation uncoupling [58]. Our result that ΔΨm of the group with melatonin treatment was slightly lower than the control group in both 143B cells and NARP cybrids was in agreement with previous studies.

In addition, the effectiveness of melatonin in cultured NARP cybrids suggests that it might have a potentially beneficial role in the treatment of patients with mitochondrial T8993G mutation. Current treatments for such patients are rather limited. Also, as stroke-like syndrome is a common clinical presentation in inherited mitochondrial disorders [59], experiments focused on of H/RO treatment in NARP cybrids might provide a better understanding of the pathophysiology of stroke-like syndrome in inherited mitochondrial disorder and provide the basis for potential treatment of in the future. 

Several recent studies have investigated the influence of H/RO on mitochondrial dynamics. These findings suggest that manipulating mitochondrial dynamics may provide a novel therapeutic strategy for cardioprotection. Giedt et al. suggested that H/RO results in increased mitochondrial fission in cultured vascular endothelial cells [60]. Ong et al. showed that inhibiting mitochondrial fission with mdivi-1 protected the heart from H/RO-induced injury by preventing the opening of the mPTP [61]. Elongated mitochondria have enhanced mitochondrial respiration capacity and hyperpolarized ΔΨm, which may be better equipped to withstand the metabolic stresses associated with H/RO injury [62,63].

In conclusion, NARP-induced inhibition of F1F0-ATPase significantly enhances apoptotic death and mitochondrial dysfunction in response to the H/RO insults. NARP-augmented apoptotic death upon H/RO insults is associated with an enhanced mROS formation, which augments the depletion of cardiolipin and retards mitochondrial movement. Melatonin significantly prevents mROS-mediated depletion of cardiolipin and mCa^2+^ accumulation in NARP cybrids in response to H/RO treatment. Melatonin improves NARP-augmented H/RO insults-induced retardation of mitochondrial movement. A better understanding of the influence of the F1F0-ATPase defect on H/RO treatment might hold great therapeutic potential for rescuing H/RO insults-induced cell damage and for NARP-induced pathologies and diseases. Furthermore, melatonin may potentially rescue patients with H/RO insults (e.g. acute myocardial infarction, cerebral infarction), even in patients associated with NARP symptoms.

## Supporting Information

Figure S1 **Quantitative analysis of the effects of H/RO (with different hypoxia durations) on mitochondrial functions in NARP cybrids and 143B cells (Figure 3).** (A) NAO (to measure cardiolipin) fluorescent intensity percentage in response to H/RO (H: 6h, RO: 2h) treatment in 143B cells, analyzed at 0, 21, 42, and 60 min after the start of recording. (B) NAO fluorescent intensity percentage in response to H/RO treatment in NARP cybrids. (C) Rhod-2 (to measure mCa^2+^) fluorescent intensity in response to H/RO treatment in 143B cells. (D) Rhod-2 fluorescent intensity in response to H/RO treatment in NARP cybrids, *P<0.05.
(TIF)

Figure S2 **Quantitative analysis of the effects of melatonin on ΔΨm and mROS upon H_2_O_2_-augmented H/RO in 143B cells and NARP cybrids (Figure 5).** (A) TMRM (to measure ΔΨm) fluorescent intensity in 143B cells in response to H_2_O_2_-augmented H/RO (H: 6h, RO: 2h) treatment, analyzed at 0, 10, 20, and 30 min after adding H_2_O_2_. (B) TMRM fluorescent intensity in NARP cybrids in response to H_2_O_2_-augmented H/RO treatment. (C) DCF (to measure mROS) fluorescent intensity in 143B cells in response to H_2_O_2_-augmented H/RO treatment. (D) DCF fluorescent intensity in NARP cybrids in response to H_2_O_2_-augmented H/RO treatment.
(TIF)

Figure S3 **Quantitative analysis of the effects of melatonin on cardiolipin and mCa^2+^ upon H_2_O_2_-augmented H/RO in 143B cells and NARP cybrids (Figure 6).** (A) NAO (to measure cardiolipin) fluorescent intensity in 143B cells in response to H_2_O_2_-augmented H/RO (H: 6h, RO: 2h) treatment, analyzed at 0, 10, 20, and 30 min after adding H_2_O_2_. (B) NAO fluorescent intensity in NARP cybrids in response to H_2_O_2_-augmented H/RO treatment. (C) Rhod-2 (to measure mCa^2+^) fluorescent intensity in 143B cells in response to H_2_O_2_-augmented H/RO treatment. (D) Rhod-2 fluorescent intensity in NARP cybrids in response to H_2_O_2_-augmented H/RO treatment. *P<0.05.
(TIF)

Figure S4 **Effects of mitochondrial specific antioxidant (MitoQ) and general antioxidant (vitamin E) on ΔΨm and mROS upon H_2_O_2_-augmented H/RO in 143B cells and NARP cybrids.** (A, B) Adding MitoQ 0.2 nM or vitamin E 200 μm during H_2_O_2_-augmented H/RO treatment effectively protected ΔΨm from depolarization in 143B cells and NARP cybrids. (C, D) Adding MitoQ 0.2 nM or vitamin E 200 μm during H_2_O_2_-augmented H/RO effectively suppressed mROS formation in 143B cells and NARP cybrids, but the effect was better in the MitoQ group. (A′-D′) Quantitative analyses of A-D. n=6. FI: fluorescence intensity. VitE : vitamin E.
(TIF)

Figure S5 **Effects of mitochondrial specific antioxidant (MitoQ) and general antioxidant (vitamin E)** **on cardiolipin and mCa^2+^ upon H_2_O_2_ –augmented H/RO in 143B cells and NARP cybrids**. (A, B) Adding MitoQ 0.2 nM or vitamin E 200 μm during H_2_O_2_-augmented H/RO treatment effectively protected cardiolipin from depletion. (C) In 143B cells, no obvious mCa^2+^ accumulation was noted in response to H_2_O_2_-augmented H/RO. (D) Adding MitoQ 0.2 nM or vitamin E 200 μm during H_2_O_2_-augmented H/RO effectively suppressed mCa^2+^accumulation. (A′-D′) Quantitative analyses of A-D. n=6. FI: fluorescence intensity. VitE : vitamin E.
(TIF)

Figure S6 **Quantitative analysis of the effects of mitochondrial specific antioxidant (MitoQ) and general antioxidant (vitamin E) on ΔΨm and mROS upon H_2_O_2_-augmented H/RO in 143B cells and NARP cybrids (Figure S4).** (A) TMRM (to measure ΔΨm) fluorescent intensity in 143B cells in response to H_2_O_2_-augmented H/RO (H: 6h, RO: 2h) treatment, analyzed at 0, 10, 20, and 30 min after adding H_2_O_2_. (B) TMRM fluorescent intensity in NARP cybrids in response to H_2_O_2_-augmented H/RO treatment. (C) DCF (to measure mROS) fluorescent intensity in 143B cells in response to H_2_O_2_-augmented H/RO treatment. (D) DCF fluorescent intensity in NARP cybrids in response to H_2_O_2_-augmented H/RO treatment. *P<0.05.
(TIF)

Figure S7 **Quantitative analysis of the effects of mitochondrial specific antioxidant (MitoQ) and general antioxidant (vitamin E) on cardiolipin and mCa^2+^ upon H_2_O_2_-augmented H/RO in 143B cells and NARP cybrids (Figure S5).** (A) NAO (to measure cardiolipin) fluorescent intensity in 143B cells in response to H_2_O_2_-augmented H/RO (H: 6h, RO: 2h) treatment, analyzed at 0, 10, 20, and 30 min after adding H_2_O_2_. (B) NAO fluorescent intensity in NARP cybrids in response to H_2_O_2_-augmented H/RO treatment. (C) Rhod-2 (to measure mCa^2+^) fluorescent intensity in 143B cells in response to H_2_O_2_-augmented H/RO treatment. (D) Rhod-2 fluorescent intensity in NARP cybrids in response to H_2_O_2_-augmented H/RO treatment. *P<0.05.
(TIF)

## References

[1] Braunwald E, Kloner RA (1985) Myocardial reperfusion: a double-edged sword? J Clin Invest 76: 1713-1719. doi:10.1172/JCI112160. PubMed: 4056048. 4056048 PMC424191

[2] Loor G, Kondapalli J, Iwase H, Chandel NS, Waypa GB et al. (2011) Mitochondrial oxidant stress triggers cell death in simulated ischemia-reperfusion. Biochim Biophys Acta 1813: 1382-1394. doi:10.1016/j.bbamcr.2010.12.008. PubMed: 21185334. 21185334 PMC3089816

[3] Abramov AY, Scorziello A, Duchen MR (2007) Three distinct mechanisms generate oxygen free radicals in neurons and contribute to cell death during anoxia and reoxygenation. J Neurosci 27: 1129-1138. doi:10.1523/JNEUROSCI.4468-06.2007. PubMed: 17267568. 17267568 PMC6673180

[4] Folbergrová J, Li PA, Uchino H, Smith ML, Siesjö BK (1997) Changes in the bioenergetic state of rat hippocampus during 2.5 min of ischemia, and prevention of cell damage by cyclosporin A in hyperglycemic subjects. Exp Brain Res 114: 44-50. doi:10.1007/PL00005622. PubMed: 9125450. 9125450

[5] Katsura K, Rodriguez de Turco EB, Folbergrová J, Bazan NG, Siesjö BK (1993) Coupling among energy failure, loss of ion homeostasis, and phospholipase A2 and C activation during ischemia. J Neurochem 61: 1677-1684. doi:10.1111/j.1471-4159.1993.tb09803.x. PubMed: 8228987. 8228987

[6] Liu RR, Murphy TH (2009) Reversible cyclosporin A-sensitive mitochondrial depolarization occurs within minutes of stroke onset in mouse somatosensory cortex in vivo: a two-photon imaging study. J Biol Chem 284: 36109-36117. doi:10.1074/jbc.M109.055301. PubMed: 19892710. 19892710 PMC2794726

[7] Perrelli MG, Pagliaro P, Penna C (2011) Ischemia/reperfusion injury and cardioprotective mechanisms: Role of mitochondria and reactive oxygen species. World. J Cardiol 3: 186-200. 10.4330/wjc.v3.i6.186 PMC3139040 21772945

[8] Sanderson TH, Reynolds CA, Kumar R, Przyklenk K, Hüttemann M (2013) Molecular mechanisms of ischemia-reperfusion injury in brain: pivotal role of the mitochondrial membrane potential in reactive oxygen species generation. Mol Neurobiol 47: 9-23. PubMed: 23011809. 23011809 10.1007/s12035-012-8344-z PMC3725766

[9] Baines CP (2009) The mitochondrial permeability transition pore and ischemia-reperfusion injury. Basic Res Cardiol 104: 181-188. doi:10.1016/j.amjcard.2009.08.522. PubMed: 19242640. 19242640 PMC2671061

[10] Griffiths EJ, Halestrap AP (1995) Mitochondrial non-specific pores remain closed during cardiac ischaemia, but open upon reperfusion. Biochem J 307 ( 1): 93-98. PubMed: 7717999. 7717999 10.1042/bj3070093 PMC1136749

[11] Halestrap AP, Clarke SJ, Javadov SA (2004) Mitochondrial permeability transition pore opening during myocardial reperfusion--a target for cardioprotection. Cardiovasc Res 61: 372-385. doi:10.1016/S0008-6363(03)00533-9. PubMed: 14962470. 14962470

[12] Murphy E, Steenbergen C (2008) Mechanisms underlying acute protection from cardiac ischemia-reperfusion injury. Physiol Rev 88: 581-609. doi:10.1152/physrev.00024.2007. PubMed: 18391174. 18391174 PMC3199571

[13] Weiss JN, Korge P, Honda HM, Ping P (2003) Role of the mitochondrial permeability transition in myocardial disease. Circ Res 93: 292-301. doi:10.1161/01.RES.0000087542.26971.D4. PubMed: 12933700. 12933700

[14] Di Lisa F, Bernardi P (2009) A CaPful of mechanisms regulating the mitochondrial permeability transition. J Mol Cell Cardiol 46: 775-780. doi:10.1016/j.yjmcc.2009.03.006. PubMed: 19303419. 19303419

[15] Di Lisa F, Canton M, Carpi A, Kaludercic N, Menabò R et al. (2011) Mitochondrial injury and protection in ischemic pre- and postconditioning. Antioxid Redox Signal 14: 881-891. doi:10.1089/ars.2010.3375. PubMed: 20615074. 20615074

[16] Heusch G, Boengler K, Schulz R (2010) Inhibition of mitochondrial permeability transition pore opening: the Holy Grail of cardioprotection. Basic Res Cardiol 105: 151-154. doi:10.1007/s00395-009-0080-9. PubMed: 20066536. 20066536

[17] Ruiz-Meana M, Abellán A, Miró-Casas E, Garcia-Dorado D (2007) Opening of mitochondrial permeability transition pore induces hypercontracture in Ca2+ overloaded cardiac myocytes. Basic Res Cardiol 102: 542-552. doi:10.1007/s00395-007-0675-y. PubMed: 17891523. 17891523

[18] Ambrosio G, Zweier JL, Duilio C, Kuppusamy P, Santoro G et al. (1993) Evidence that mitochondrial respiration is a source of potentially toxic oxygen free radicals in intact rabbit hearts subjected to ischemia and reflow. J Biol Chem 268: 18532-18541. PubMed: 8395507. 8395507

[19] Bolli R, Marbán E (1999) Molecular and cellular mechanisms of myocardial stunning. Physiol Rev 79: 609-634. PubMed: 10221990. 10221990 10.1152/physrev.1999.79.2.609

[20] Baracca A, Barogi S, Carelli V, Lenaz G, Solaini G (2000) Catalytic activities of mitochondrial ATP synthase in patients with mitochondrial DNA T8993G mutation in the ATPase 6 gene encoding subunit a. J Biol Chem 275: 4177-4182. doi:10.1074/jbc.275.6.4177. PubMed: 10660580. 10660580

[21] Peng TI, Hsiao CW, Reiter RJ, Tanaka M, Lai YK et al. (2012) mtDNA T8993G mutation-induced mitochondrial complex V inhibition augments cardiolipin-dependent alterations in mitochondrial dynamics during oxidative, Ca(2+), and lipid insults in NARP cybrids: a potential therapeutic target for melatonin. J Pineal Res 52: 93-106. doi:10.1111/j.1600-079X.2011.00923.x. PubMed: 21812817. 21812817

[22] Geromel V, Kadhom N, Cebalos-Picot I, Ouari O, Polidori A et al. (2001) Superoxide-induced massive apoptosis in cultured skin fibroblasts harboring the neurogenic ataxia retinitis pigmentosa (NARP) mutation in the ATPase-6 gene of the mitochondrial DNA. Hum Mol Genet 10: 1221-1228. doi:10.1093/hmg/10.11.1221. PubMed: 11371515. 11371515

[23] Kaneko S, Okumura K, Numaguchi Y, Matsui H, Murase K et al. (2000) Melatonin scavenges hydroxyl radical and protects isolated rat hearts from ischemic reperfusion injury. Life Sci 67: 101-112. doi:10.1016/S0024-3205(00)00607-X. PubMed: 10901278. 10901278

[24] Reiter RJ, Tan DX (2003) Melatonin: a novel protective agent against oxidative injury of the ischemic/reperfused heart. Cardiovasc Res 58: 10-19. doi:10.1016/S0008-6363(02)00827-1. PubMed: 12667942. 12667942

[25] Salie R, Harper I, Cillie C, Genade S, Huisamen B et al. (2001) Melatonin protects against ischaemic-reperfusion myocardial damage. J Mol Cell Cardiol 33: 343-357. doi:10.1006/jmcc.2000.1306. PubMed: 11162138. 11162138

[26] Tan DX, Manchester LC, Reiter RJ, Qi W, Kim SJ et al. (1998) Ischemia/reperfusion-induced arrhythmias in the isolated rat heart: prevention by melatonin. J Pineal Res 25: 184-191. doi:10.1111/j.1600-079X.1998.tb00558.x. PubMed: 9745988. 9745988

[27] Tengattini S, Reiter RJ, Tan DX, Terron MP, Rodella LF et al. (2008) Cardiovascular diseases: protective effects of melatonin. J Pineal Res 44: 16-25. PubMed: 18078444. 18078444 10.1111/j.1600-079X.2007.00518.x

[28] Kilic E, Kilic U, Reiter RJ, Bassetti CL, Hermann DM (2005) Tissue-plasminogen activator-induced ischemic brain injury is reversed by melatonin: role of iNOS and Akt. J Pineal Res 39: 151-155. doi:10.1111/j.1600-079X.2005.00228.x. PubMed: 16098092. 16098092

[29] Kilic E, Kilic U, Yulug B, Hermann DM, Reiter RJ (2004) Melatonin reduces disseminate neuronal death after mild focal ischemia in mice via inhibition of caspase-3 and is suitable as an add-on treatment to tissue-plasminogen activator. J Pineal Res 36: 171-176. doi:10.1046/j.1600-079X.2003.00115.x. PubMed: 15009507. 15009507

[30] Tanaka M, Borgeld HJ, Zhang J, Muramatsu S, Gong JS et al. (2002) Gene therapy for mitochondrial disease by delivering restriction endonuclease SmaI into mitochondria. J Biomed Sci 9: 534-541. doi:10.1159/000064726. PubMed: 12372991. 12372991

[31] Mosmann T (1983) Rapid colorimetric assay for cellular growth and survival: application to proliferation and cytotoxicity assays. J Immunol Methods 65: 55-63. doi:10.1016/0022-1759(83)90303-4. PubMed: 6606682. 6606682

[32] Strober W (2001) Trypan blue exclusion test of cell viability. Curr Protoc Immunol Appendix 3: Appendix 3B 10.1002/0471142735.ima03bs21 18432654

[33] van Engeland M, Nieland LJ, Ramaekers FC, Schutte B, Reutelingsperger CP (1998) Annexin V-affinity assay: a review on an apoptosis detection system based on phosphatidylserine exposure. Cytometry 31: 1-9. doi:10.1002/(SICI)1097-0320(19980101)31:1. PubMed: 9450519. 9450519

[34] Baracca A, Sgarbi G, Mattiazzi M, Casalena G, Pagnotta E et al. (2007) Biochemical phenotypes associated with the mitochondrial ATP6 gene mutations at nt8993. Biochim Biophys Acta 1767: 913-919. doi:10.1016/j.bbabio.2007.05.005. PubMed: 17568559. 17568559

[35] Lebiedzinska M, Karkucinska-Wieckowska A, Wojtala A, Suski JM, Szabadkai G et al. (2013) Disrupted ATP synthase activity and mitochondrial hyperpolarisation-dependent oxidative stress is associated with p66Shc phosphorylation in fibroblasts of NARP patients. Int J Biochem Cell Biol 45: 141-150. doi:10.1016/j.biocel.2012.07.020. PubMed: 22885148. 22885148

[36] Huang WY, Weng WC, Peng TI, Chien YY, Wu CL et al. (2012) Association of hyponatremia in acute stroke stage with three-year mortality in patients with first-ever ischemic stroke. Cerebrovasc Dis 34: 55-62. doi:10.1159/000338906. PubMed: 22759703. 22759703

[37] Jou MJ, Peng TI, Yu PZ, Jou SB, Reiter RJ et al. (2007) Melatonin protects against common deletion of mitochondrial DNA-augmented mitochondrial oxidative stress and apoptosis. J Pineal Res 43: 389-403. doi:10.1111/j.1600-079X.2007.00490.x. PubMed: 17910608. 17910608

[38] Peng TI, Yu PR, Chen JY, Wang HL, Wu HY et al. (2006) Visualizing common deletion of mitochondrial DNA-augmented mitochondrial reactive oxygen species generation and apoptosis upon oxidative stress. Biochim Biophys Acta 1762: 241-255. doi:10.1016/j.bbadis.2005.10.008. PubMed: 16368227. 16368227

[39] Mattiazzi M, Vijayvergiya C, Gajewski CD, DeVivo DC, Lenaz G et al. (2004) The mtDNA T8993G (NARP) mutation results in an impairment of oxidative phosphorylation that can be improved by antioxidants. Hum Mol Genet 13: 869-879. doi:10.1093/hmg/ddh103. PubMed: 14998933. 14998933

[40] Lesnefsky EJ, Chen Q, Slabe TJ, Stoll MS, Minkler PE et al. (2004) Ischemia, rather than reperfusion, inhibits respiration through cytochrome oxidase in the isolated, perfused rabbit heart: role of cardiolipin. Am J Physiol Heart Circ Physiol 287: H258-H267. doi:10.1152/ajpheart.00348.2003. PubMed: 14988071. 14988071

[41] Paradies G, Petrosillo G, Pistolese M, Di Venosa N, Serena D et al. (1999) Lipid peroxidation and alterations to oxidative metabolism in mitochondria isolated from rat heart subjected to ischemia and reperfusion. Free Radic Biol Med 27: 42-50. doi:10.1016/S0891-5849(99)90636-6. PubMed: 10443918. 10443918

[42] Petrosillo G, Ruggiero FM, Di Venosa N, Paradies G (2003) Decreased complex III activity in mitochondria isolated from rat heart subjected to ischemia and reperfusion: role of reactive oxygen species and cardiolipin. FASEB J 17: 714-716. PubMed: 12586737. 12586737 10.1096/fj.02-0729fje

[43] Paradies G, Petrosillo G, Pistolese M, Di Venosa N, Federici A et al. (2004) Decrease in mitochondrial complex I activity in ischemic/reperfused rat heart: involvement of reactive oxygen species and cardiolipin. Circ Res 94: 53-59. doi:10.1161/01.RES.0000109416.56608.64. PubMed: 14656928. 14656928

[44] Petrosillo G, Casanova G, Matera M, Ruggiero FM, Paradies G (2006) Interaction of peroxidized cardiolipin with rat-heart mitochondrial membranes: induction of permeability transition and cytochrome c release. FEBS Lett 580: 6311-6316. doi:10.1016/j.febslet.2006.10.036. PubMed: 17083938. 17083938

[45] Petrosillo G, Moro N, Ruggiero FM, Paradies G (2009) Melatonin inhibits cardiolipin peroxidation in mitochondria and prevents the mitochondrial permeability transition and cytochrome c release. Free Radic Biol Med 47: 969-974. doi:10.1016/j.freeradbiomed.2009.06.032. PubMed: 19577639. 19577639

[46] Venegas C, García JA, Escames G, Ortiz F, López A et al. (2012) Extrapineal melatonin: analysis of its subcellular distribution and daily fluctuations. J Pineal Res 52: 217-227. doi:10.1111/j.1600-079X.2011.00931.x. PubMed: 21884551. 21884551

[47] Rosen J, Than NN, Koch D, Poeggeler B, Laatsch H et al. (2006) Interactions of melatonin and its metabolites with the ABTS cation radical: extension of the radical scavenger cascade and formation of a novel class of oxidation products, C2-substituted 3-indolinones. J Pineal Res 41: 374-381. doi:10.1111/j.1600-079X.2006.00379.x. PubMed: 17014695. 17014695

[48] Tan DX, Manchester LC, Reiter RJ, Qi WB, Karbownik M et al. (2000) Significance of melatonin in antioxidative defense system: reactions and products. Biol Signals Recept 9: 137-159. doi:10.1159/000014635. PubMed: 10899700. 10899700

[49] Tan DX, Manchester LC, Terron MP, Flores LJ, Reiter RJ (2007) One molecule, many derivatives: a never-ending interaction of melatonin with reactive oxygen and nitrogen species? J Pineal Res 42: 28-42. doi:10.1111/j.1600-079X.2006.00407.x. PubMed: 17198536. 17198536

[50] Galano A, Tan DX, Reiter RJ (2011) Melatonin as a natural ally against oxidative stress: a physicochemical examination. J Pineal Res 51: 1-16. doi:10.1111/j.1600-079X.2011.00916.x. PubMed: 21752095. 21752095

[51] Galano A, Tan DX, Reiter RJ (2013) On the free radical scavenging activities of melatonin's metabolites, AFMK and AMK. J Pineal Res 54: 245-257. doi:10.1111/jpi.12010. PubMed: 22998574. 22998574

[52] Rodriguez C, Mayo JC, Sainz RM, Antolín I, Herrera F et al. (2004) Regulation of antioxidant enzymes: a significant role for melatonin. J Pineal Res 36: 1-9. doi:10.1046/j.1600-079X.2003.00092.x. PubMed: 14675124. 14675124

[53] Drew JE, Barrett P, Mercer JG, Moar KM, Canet E et al. (2001) Localization of the melatonin-related receptor in the rodent brain and peripheral tissues. J Neuroendocrinol 13: 453-458. doi:10.1046/j.1365-2826.2001.00651.x. PubMed: 11328456. 11328456

[54] Wang X, Sirianni A, Pei Z, Cormier K, Smith K et al. (2011) The melatonin MT1 receptor axis modulates mutant Huntingtin-mediated toxicity. J Neurosci 31: 14496-14507. doi:10.1523/JNEUROSCI.3059-11.2011. PubMed: 21994366. 21994366 PMC3213696

[55] Peyrot F, Ducrocq C (2008) Potential role of tryptophan derivatives in stress responses characterized by the generation of reactive oxygen and nitrogen species. J Pineal Res 45: 235-246. doi:10.1111/j.1600-079X.2008.00580.x. PubMed: 18341517. 18341517

[56] Andrabi SA, Sayeed I, Siemen D, Wolf G, Horn TF (2004) Direct inhibition of the mitochondrial permeability transition pore: a possible mechanism responsible for anti-apoptotic effects of melatonin. FASEB J 18: 869-871. PubMed: 15033929. 15033929 10.1096/fj.03-1031fje

[57] Petrosillo G, Colantuono G, Moro N, Ruggiero FM, Tiravanti E et al. (2009) Melatonin protects against heart ischemia-reperfusion injury by inhibiting mitochondrial permeability transition pore opening. Am J Physiol Heart Circ Physiol 297: H1487-H1493. doi:10.1152/ajpheart.00163.2009. PubMed: 19684190. 19684190

[58] Tan DX, Manchester LC, Liu X, Rosales-Corral SA, Acuna-Castroviejo D et al. (2013) Mitochondria and chloroplasts as the original sites of melatonin synthesis: a hypothesis related to melatonin's primary function and evolution in eukaryotes. J Pineal Res 54: 127-138. doi:10.1111/jpi.12026. PubMed: 23137057. 23137057

[59] Finsterer J (2012) Inherited mitochondrial disorders. Adv Exp Med Biol 942: 187-213. doi:10.1007/978-94-007-2869-1_8. PubMed: 22399423. 22399423

[60] Giedt RJ, Yang C, Zweier JL, Matzavinos A, Alevriadou BR (2012) Mitochondrial fission in endothelial cells after simulated ischemia/reperfusion: role of nitric oxide and reactive oxygen species. Free Radic Biol Med 52: 348-356. doi:10.1016/j.freeradbiomed.2011.10.491. PubMed: 22100972. 22100972 PMC3253175

[61] Ong SB, Subrayan S, Lim SY, Yellon DM, Davidson SM et al. (2010) Inhibiting mitochondrial fission protects the heart against ischemia/reperfusion injury. Circulation 121: 2012-2022. doi:10.1161/CIRCULATIONAHA.109.906610. PubMed: 20421521. 20421521

[62] Chen H, Chomyn A, Chan DC (2005) Disruption of fusion results in mitochondrial heterogeneity and dysfunction. J Biol Chem 280: 26185-26192. doi:10.1074/jbc.M503062200. PubMed: 15899901. 15899901

[63] Ong SB, Hausenloy DJ (2010) Mitochondrial morphology and cardiovascular disease. Cardiovasc Res 88: 16-29. doi:10.1093/cvr/cvq237. PubMed: 20631158. 20631158 PMC2936127

