# Correction: mtDNA T8993G Mutation-Induced F1F0-ATP Synthase Defect Augments Mitochondrial Dysfunction Associated with hypoxia/reoxygenation: The Protective Role of Melatonin

**DOI:** 10.1371/annotation/c85feb29-046d-45f6-ba12-510f9e1d9412

**Published:** 2014-01-06

**Authors:** Wen-Yi Huang, Mei-Jie Jou, I. Peng Tsung

The name of the first author is incorrect. The correct name is: Tsung-I Peng. The correct Citation is: 

Huang W-Y, Jou M-J, Peng T-I (2013) mtDNA T8993G Mutation-Induced F1F0-ATP Synthase Defect Augments Mitochondrial Dysfunction Associated with hypoxia/reoxygenation: The Protective Role of Melatonin. PLoS ONE 8(11): e81546. doi:10.1371/journal.pone.0081546.

The first and third authors were incorrectly indicated as having contributed equally to the article. Instead, the first and second authors, Wen-Yi Huang and Mei-Jie Jou, should be indicated as having contributed equally to the article.

In addition, the last author Tsung-I Peng was not correctly indicated as the Corresponding Author of the article. Prof. Peng's email address is: tipeng@cgmh.org.tw.

