# Supplementary figures and images for: mtDNA T8993G Mutation-Induced F1F0-ATP Synthase Defect Augments Mitochondrial Dysfunction Associated with hypoxia/reoxygenation: The Protective Role of Melatonin
Source: PLoS One. 2013 Nov 29;8(11):e81546. doi: 10.1371/journal.pone.0081546 (PMC3843685; doi:10.1371/journal.pone.0081546)

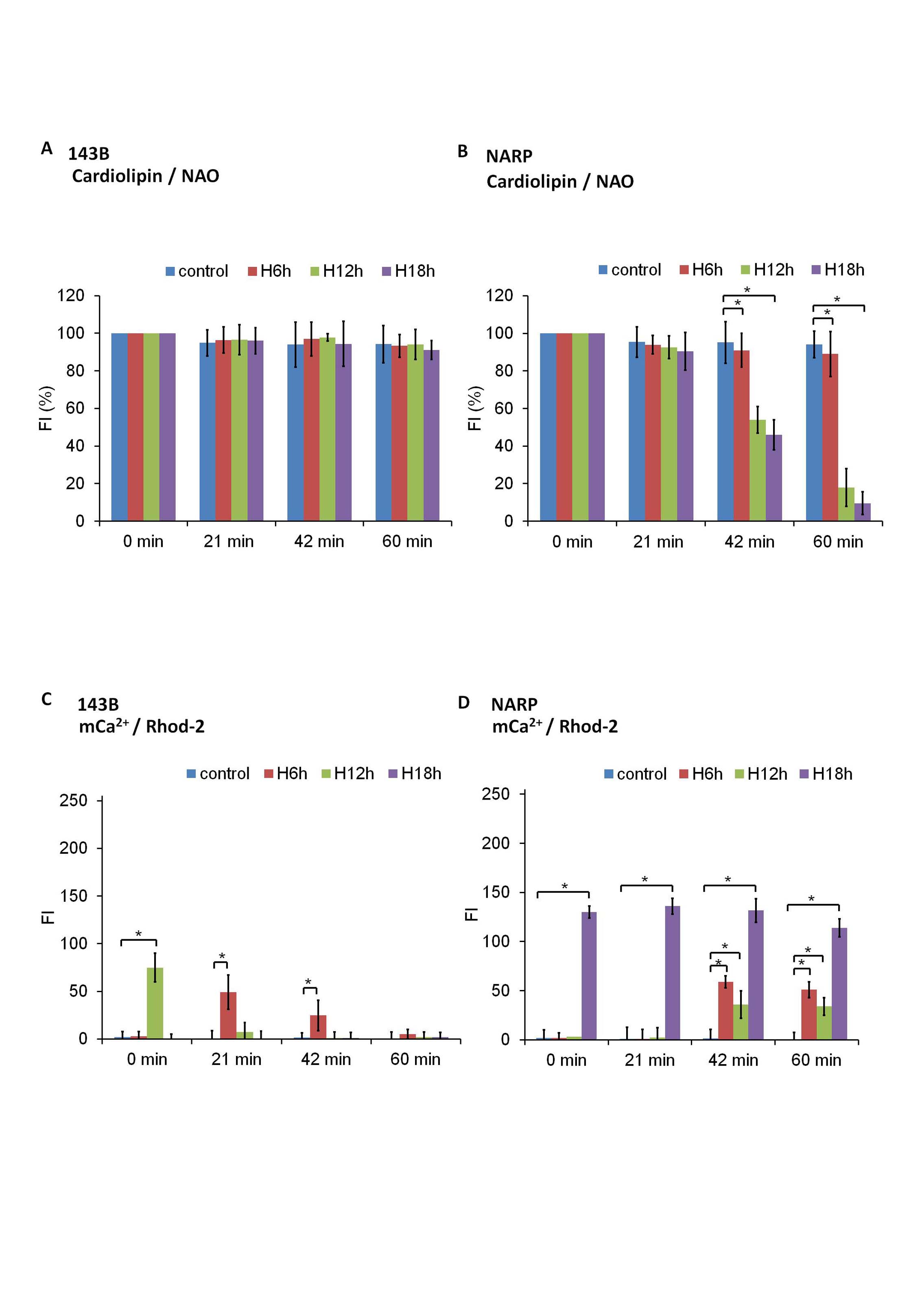

Supplement: Figure S1 — Quantitative analysis of the effects of H/RO (with different hypoxia durations) on mitochondrial functions in NARP cybrids and 143B cells (Figure 3). (A) NAO (to measure cardiolipin) fluorescent intensity percentage in response to H/RO (H: 6h, RO: 2h) treatment in 143B cells, analyzed at 0, 21, 42, and 60 min after the start of recording. (B) NAO fluorescent intensity percentage in response to H/RO treatment in NARP cybrids. (C) Rhod-2 (to measure mCa2+) fluorescent intensity in response to H/RO treatment in 143B cells. (D) Rhod-2 fluorescent intensity in response to H/RO treatment in NARP cybrids, *P<0.05. (TIF) [file pone.0081546.s001.tif]

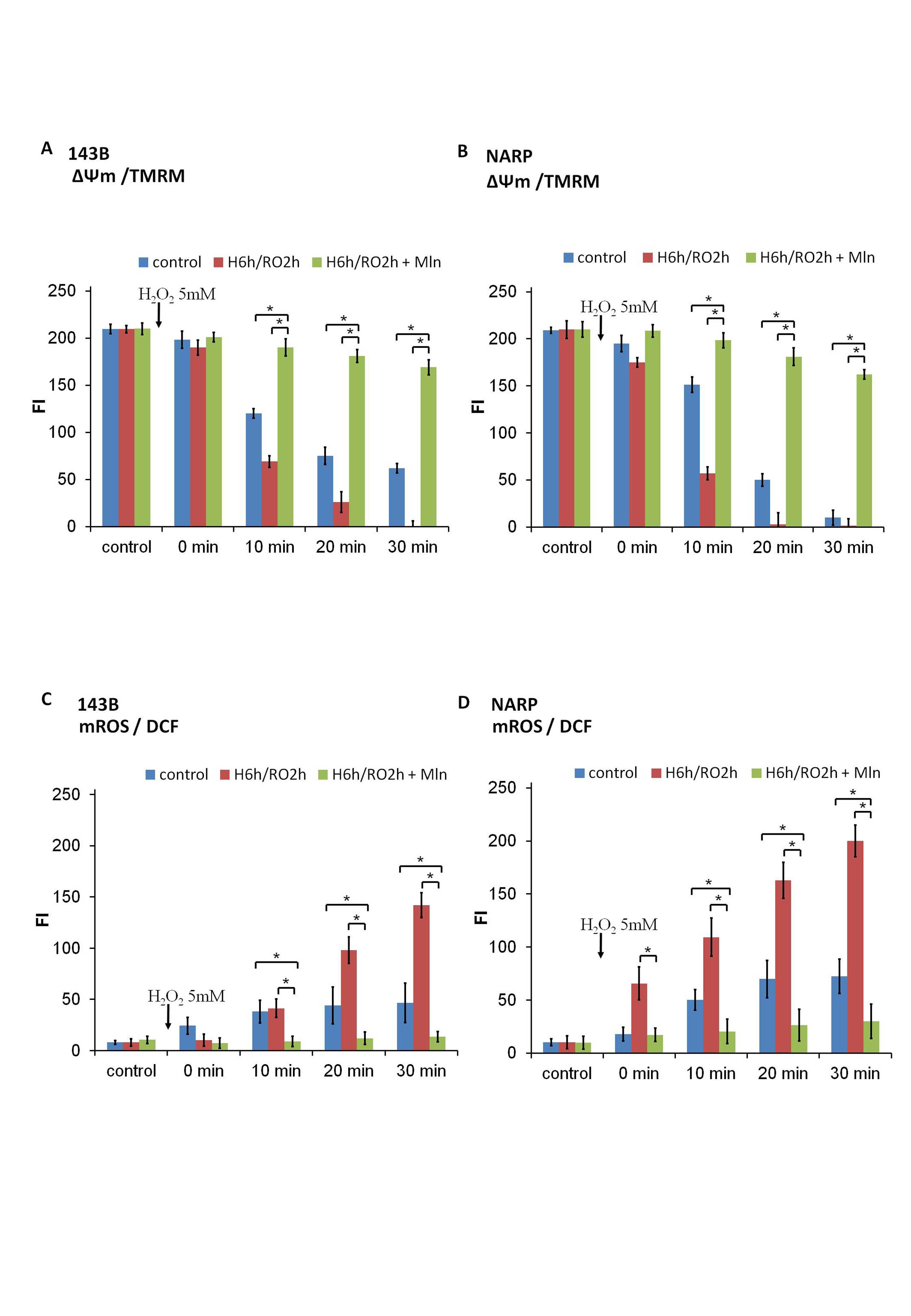

Supplement: Figure S2 — Quantitative analysis of the effects of melatonin on ΔΨm and mROS upon H2O2-augmented H/RO in 143B cells and NARP cybrids (Figure 5). (A) TMRM (to measure ΔΨm) fluorescent intensity in 143B cells in response to H2O2-augmented H/RO (H: 6h, RO: 2h) treatment, analyzed at 0, 10, 20, and 30 min after adding H2O2. (B) TMRM fluorescent intensity in NARP cybrids in response to H2O2-augmented H/RO treatment. (C) DCF (to measure mROS) fluorescent intensity in 143B cells in response to H2O2-augmented H/RO treatment. (D) DCF fluorescent intensity in NARP cybrids in response to H2O2-augmented H/RO treatment. (TIF) [file pone.0081546.s002.tif]

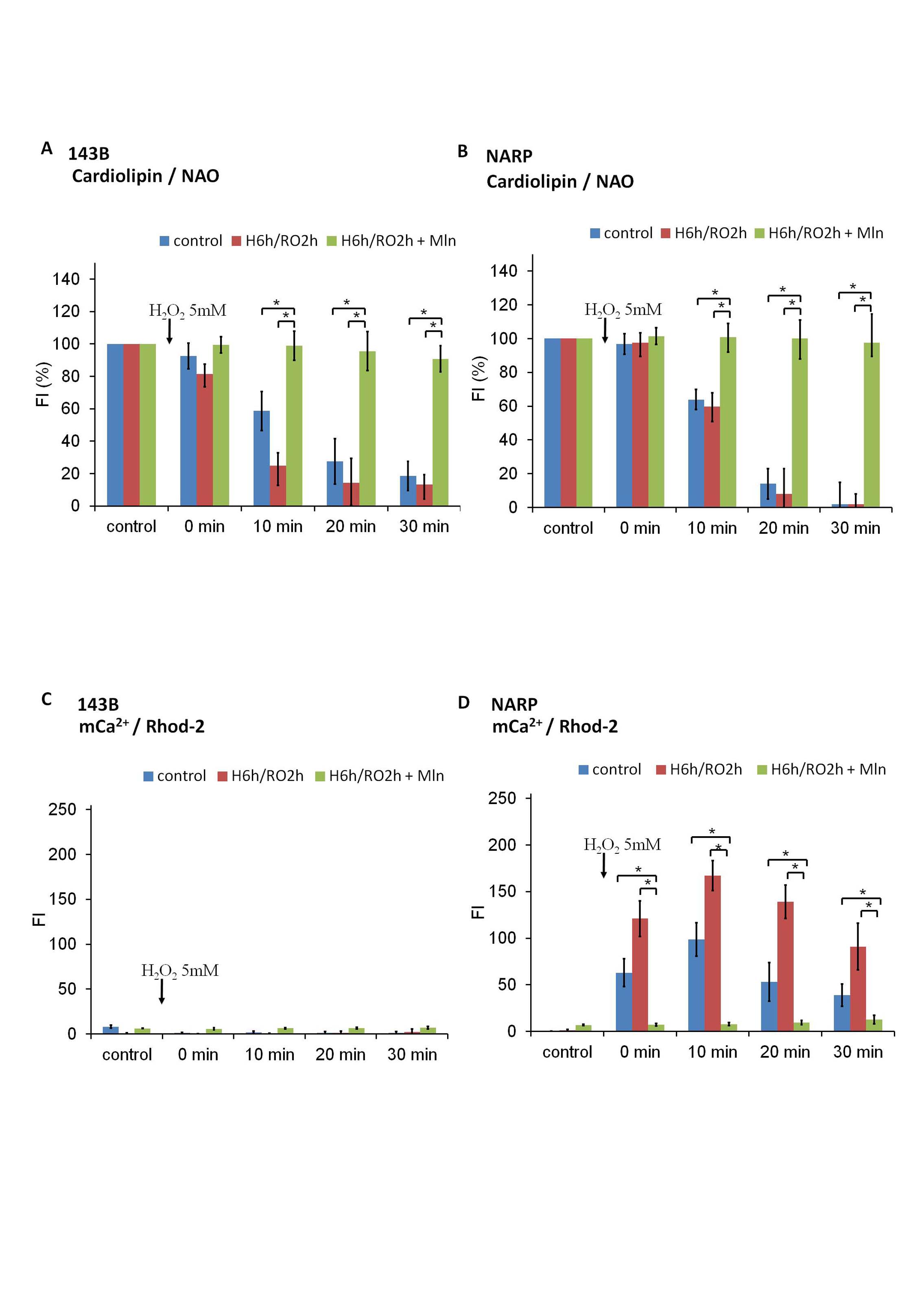

Supplement: Figure S3 — Quantitative analysis of the effects of melatonin on cardiolipin and mCa2+ upon H2O2-augmented H/RO in 143B cells and NARP cybrids (Figure 6). (A) NAO (to measure cardiolipin) fluorescent intensity in 143B cells in response to H2O2-augmented H/RO (H: 6h, RO: 2h) treatment, analyzed at 0, 10, 20, and 30 min after adding H2O2. (B) NAO fluorescent intensity in NARP cybrids in response to H2O2-augmented H/RO treatment. (C) Rhod-2 (to measure mCa2+) fluorescent intensity in 143B cells in response to H2O2-augmented H/RO treatment. (D) Rhod-2 fluorescent intensity in NARP cybrids in response to H2O2-augmented H/RO treatment. *P<0.05. (TIF) [file pone.0081546.s003.tif]

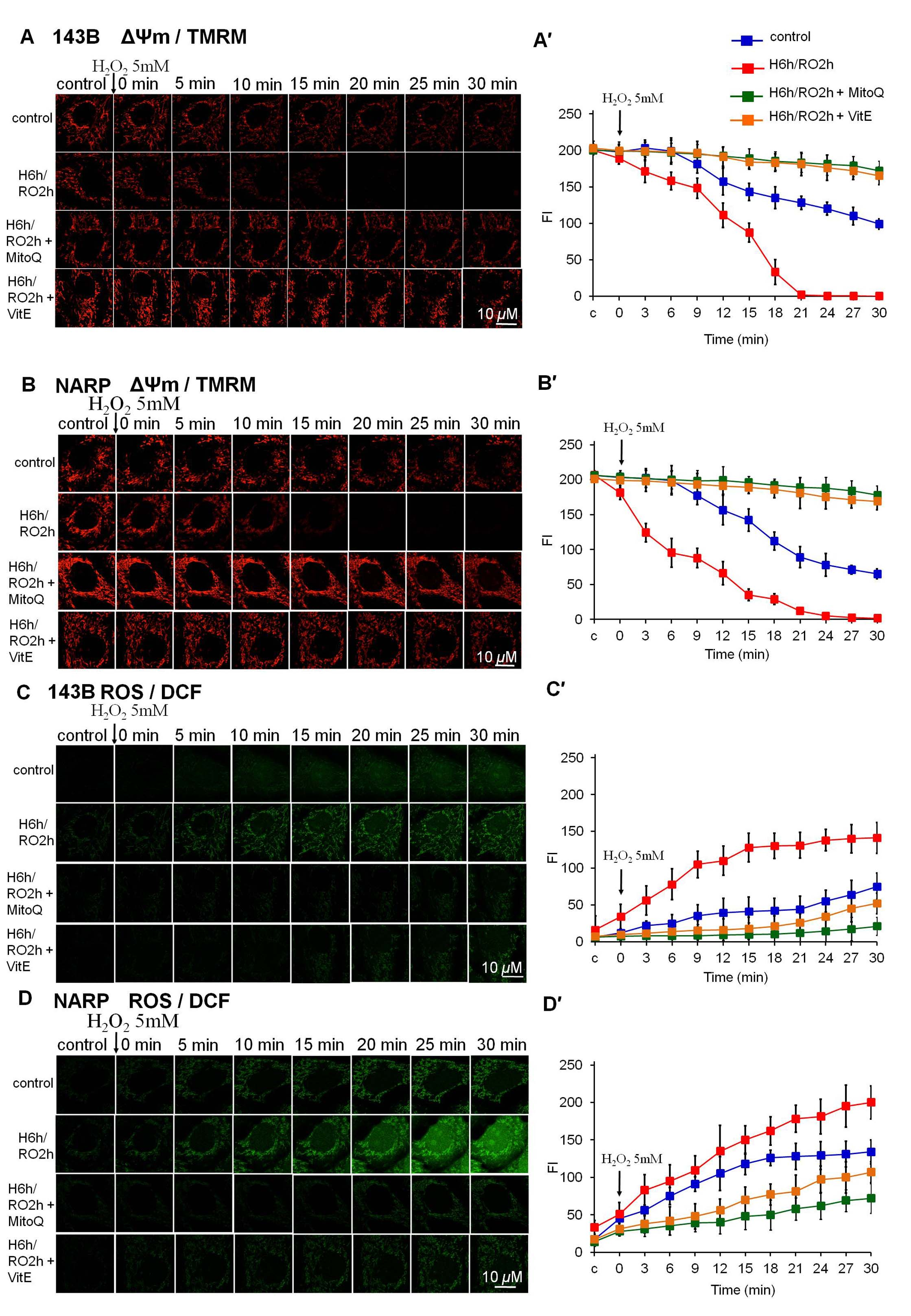

Supplement: Figure S4 — Effects of mitochondrial specific antioxidant (MitoQ) and general antioxidant (vitamin E) on ΔΨm and mROS upon H2O2-augmented H/RO in 143B cells and NARP cybrids. (A, B) Adding MitoQ 0.2 nM or vitamin E 200 μm during H2O2-augmented H/RO treatment effectively protected ΔΨm from depolarization in 143B cells and NARP cybrids. (C, D) Adding MitoQ 0.2 nM or vitamin E 200 μm during H2O2-augmented H/RO effectively suppressed mROS formation in 143B cells and NARP cybrids, but the effect was better in the MitoQ group. (A′-D′) Quantitative analyses of A-D. n=6. FI: fluorescence intensity. VitE : vitamin E. (TIF) [file pone.0081546.s004.tif]

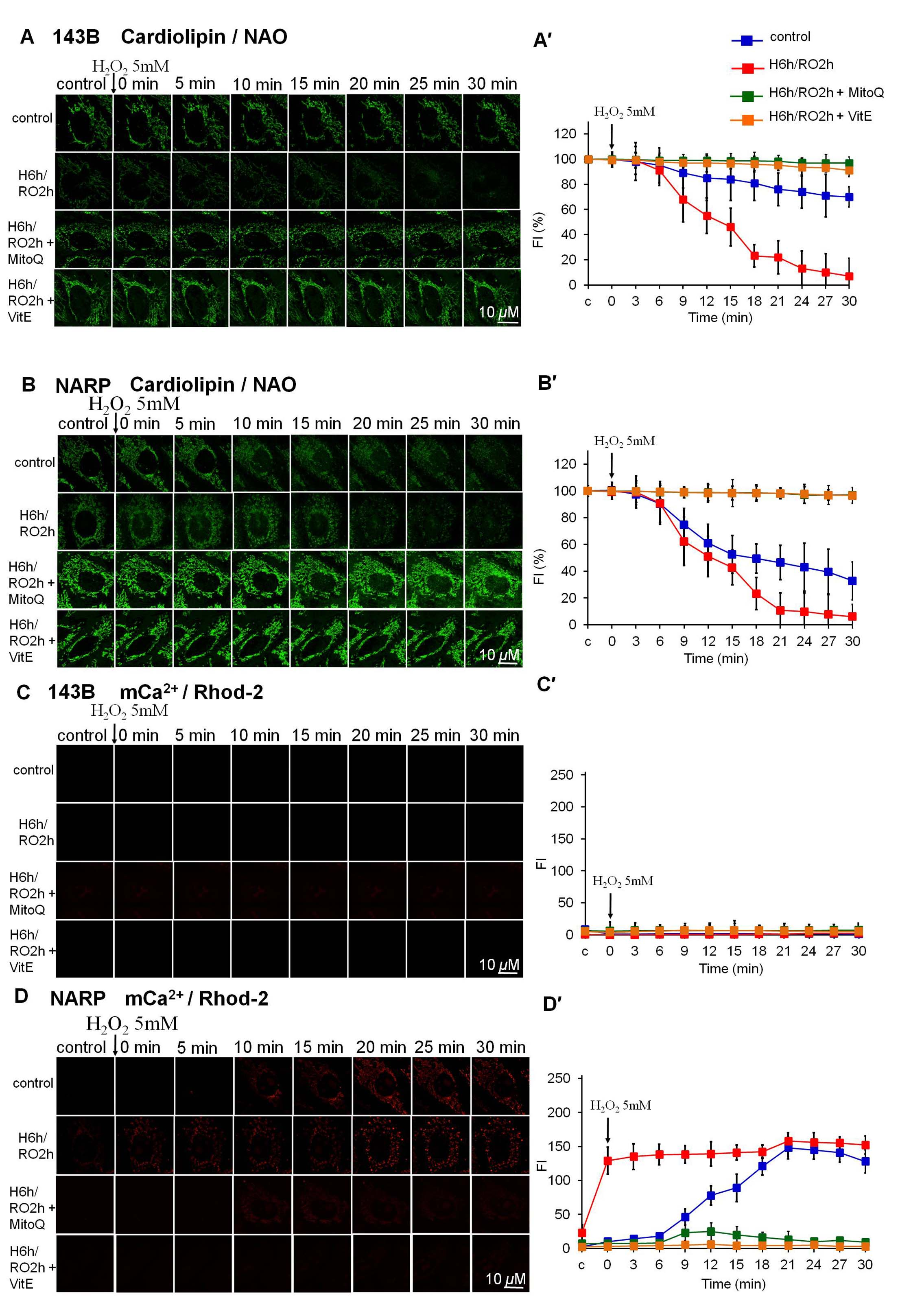

Supplement: Figure S5 — Effects of mitochondrial specific antioxidant (MitoQ) and general antioxidant (vitamin E) on cardiolipin and mCa2+ upon H2O2 –augmented H/RO in 143B cells and NARP cybrids. (A, B) Adding MitoQ 0.2 nM or vitamin E 200 μm during H2O2-augmented H/RO treatment effectively protected cardiolipin from depletion. (C) In 143B cells, no obvious mCa2+ accumulation was noted in response to H2O2-augmented H/RO. (D) Adding MitoQ 0.2 nM or vitamin E 200 μm during H2O2-augmented H/RO effectively suppressed mCa2+accumulation. (A′-D′) Quantitative analyses of A-D. n=6. FI: fluorescence intensity. VitE : vitamin E. (TIF) [file pone.0081546.s005.tif]

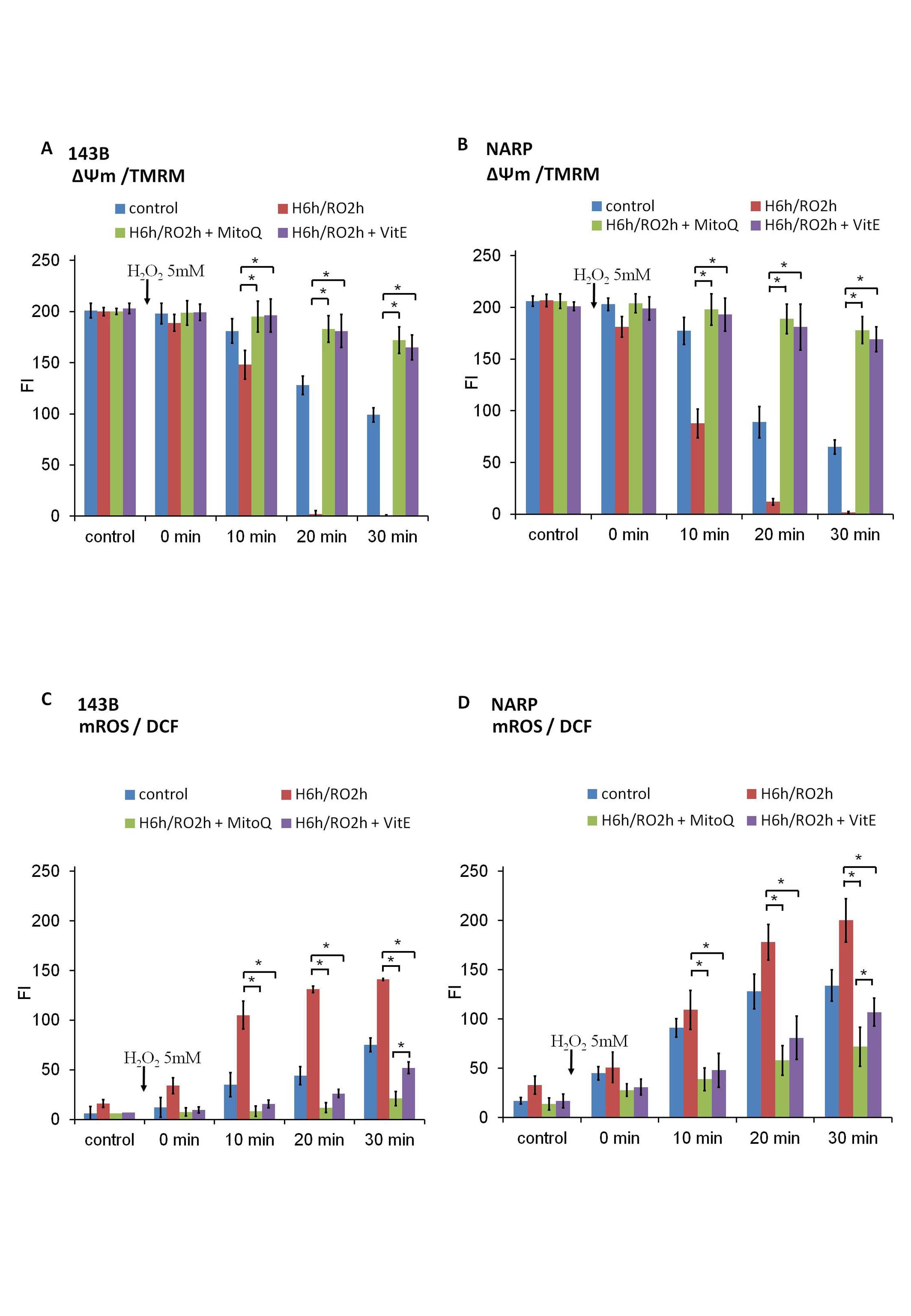

Supplement: Figure S6 — Quantitative analysis of the effects of mitochondrial specific antioxidant (MitoQ) and general antioxidant (vitamin E) on ΔΨm and mROS upon H2O2-augmented H/RO in 143B cells and NARP cybrids (Figure S4). (A) TMRM (to measure ΔΨm) fluorescent intensity in 143B cells in response to H2O2-augmented H/RO (H: 6h, RO: 2h) treatment, analyzed at 0, 10, 20, and 30 min after adding H2O2. (B) TMRM fluorescent intensity in NARP cybrids in response to H2O2-augmented H/RO treatment. (C) DCF (to measure mROS) fluorescent intensity in 143B cells in response to H2O2-augmented H/RO treatment. (D) DCF fluorescent intensity in NARP cybrids in response to H2O2-augmented H/RO treatment. *P<0.05. (TIF) [file pone.0081546.s006.tif]

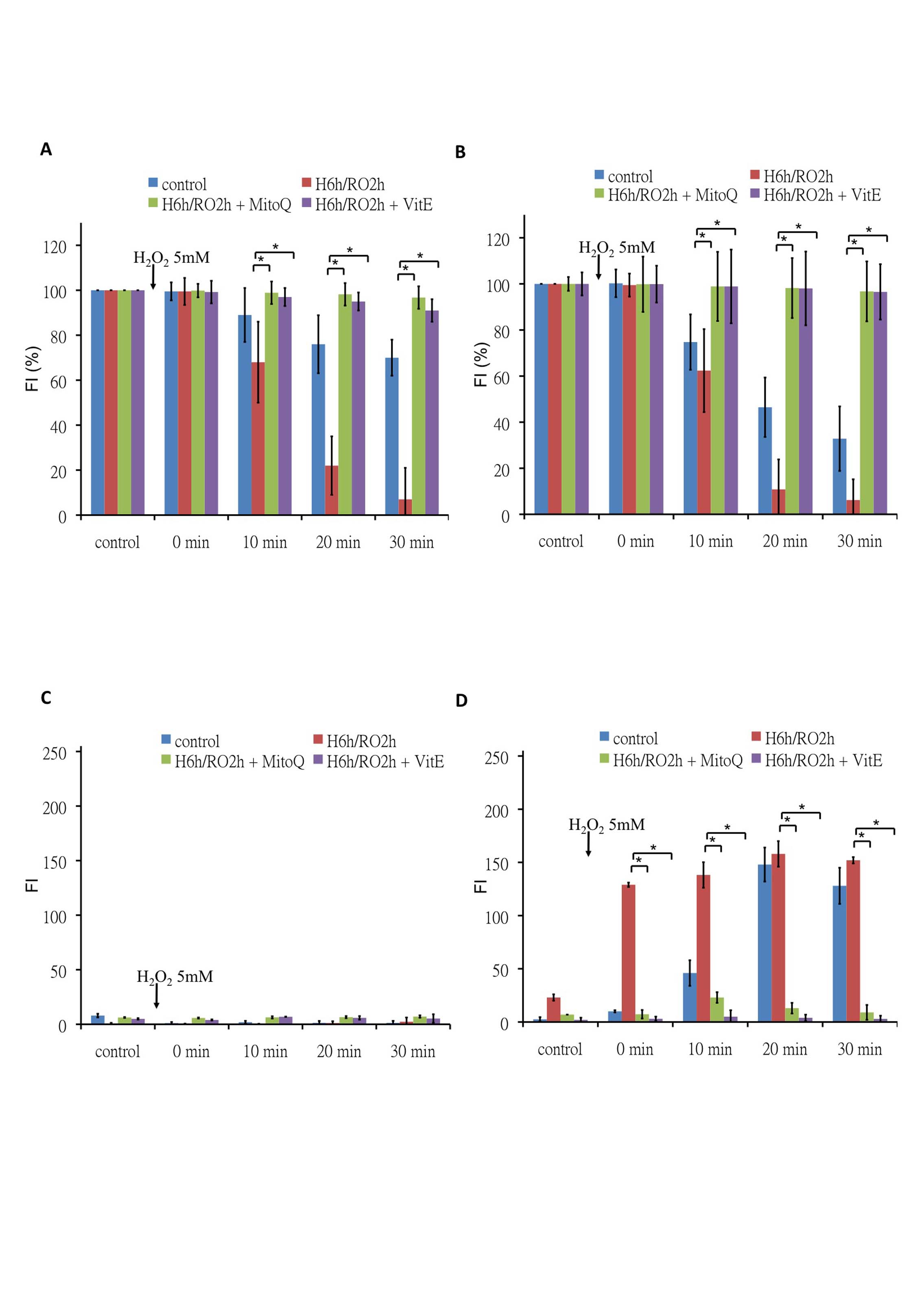

Supplement: Figure S7 — Quantitative analysis of the effects of mitochondrial specific antioxidant (MitoQ) and general antioxidant (vitamin E) on cardiolipin and mCa2+ upon H2O2-augmented H/RO in 143B cells and NARP cybrids (Figure S5). (A) NAO (to measure cardiolipin) fluorescent intensity in 143B cells in response to H2O2-augmented H/RO (H: 6h, RO: 2h) treatment, analyzed at 0, 10, 20, and 30 min after adding H2O2. (B) NAO fluorescent intensity in NARP cybrids in response to H2O2-augmented H/RO treatment. (C) Rhod-2 (to measure mCa2+) fluorescent intensity in 143B cells in response to H2O2-augmented H/RO treatment. (D) Rhod-2 fluorescent intensity in NARP cybrids in response to H2O2-augmented H/RO treatment. *P<0.05. (TIF) [file pone.0081546.s007.tif]
